# Music Therapy in Infancy and Neurodevelopmental Outcomes in Preterm Children: A Secondary Analysis of the LongSTEP Randomized Clinical Trial

**DOI:** 10.1001/jamanetworkopen.2024.10721

**Published:** 2024-05-16

**Authors:** Łucja Bieleninik, Ingrid Kvestad, Christian Gold, Andreas Størksen Stordal, Jörg Assmus, Shmuel Arnon, Cochavit Elefant, Mark Ettenberger, Tora Söderström Gaden, Dafna Haar-Shamir, Tonje Håvardstun, Marcela Lichtensztejn, Julie Mangersnes, Anne-Marthe Nygård Wiborg, Bente Johanne Vederhus, Claire M. Ghetti

**Affiliations:** 1 GAMUT—The Grieg Academy Music Therapy Research Centre, NORCE Norwegian Research Centre AS, Bergen, Norway; 2 Department of Clinical and Health Psychology, Institute of Psychology, Faculty of Social Sciences, University of Gdańsk, Gdańsk, Poland; 3 Regional Centre for Child and Youth Mental Health and Child Welfare, NORCE Norwegian Research Centre AS, Bergen, Norway; 4 Department of Clinical and Health Psychology, University of Vienna, Vienna, Austria; 5 NORCE Energy, NORCE Norwegian Research Centre AS, Bergen, Norway; 6 Department of Mathematics, University of Bergen, Bergen, Norway; 7 Meir Medical Center, Kfar-Saba, Israel; 8 Faculty of Medicine, Tel Aviv University, Tel Aviv, Israel; 9 University of Haifa, Haifa, Israel; 10 Hospital Universitario Fundación Santa Fe de Bogotá, Bogotá, Colombia; 11 Clínica de la Mujer, Bogotá, Colombia; 12 Department of Children and Youth Clinic, Haukeland University Hospital, Bergen, Norway; 13 Facultad de Ciencias de la Salud, Universidad de Ciencias Empresariales y Sociales, Buenos Aires, Argentina; 14 Oslo University Hospital, Oslo, Norway; 15 GAMUT—The Grieg Academy, Department of Music, University of Bergen, Bergen, Norway

## Abstract

**Importance:**

Preterm children are at risk for neurodevelopment impairments.

**Objective:**

To evaluate the effect of a music therapy (MT) intervention (parent-led, infant-directed singing) for premature children during the neonatal intensive care unit (NICU) stay and/or after hospital discharge on language development at 24 months’ corrected age (CA).

**Design, Setting, and Participants:**

This predefined secondary analysis followed participants in the LongSTEP (Longitudinal Study of Music Therapy's Effectiveness for Premature Infants and Their Caregivers) randomized clinical trial, which was conducted from August 2018 to April 2022 in 8 NICUs across 5 countries (Argentina, Colombia, Israel, Norway, and Poland) and included clinic follow-up visits and extended interventions after hospital discharge.

**Intervention:**

Participants were children born preterm (<35 weeks’ gestation) and their parents. Participants were randomized at enrollment to MT with standard care (SC) or SC alone; they were randomized to MT or SC again at discharge. The MT was parent-led, infant-directed singing tailored to infant responses and supported by a music therapist and was provided 3 times weekly in the NICU and/or in 7 sessions across 6 months after discharge. The SC consisted of early intervention methods of medical, nursing, and social services, without MT.

**Main Outcome and Measures:**

Primary outcome was language development, as measured by the Bayley Scales of Infant and Toddler Development, Third Edition (BSID-III) language composite score, with the remaining BSID-III composite and subscale scores as the secondary outcomes. Group differences in treatment effects were assessed using linear mixed-effects models using all available data.

**Results:**

Of 206 participants (103 female infants [50%]; mean [SD] GA, 30.5 [2.7] weeks), 51 were randomized to MT and 53 to SC at enrollment; at discharge, 52 were randomized to MT and 50 to SC. A total of 112 (54%) were retained at the 24 months’ CA follow-up. Most participants (79 [70%] to 93 [83%]) had BSID-III scores in the normal range (≥85). Mean differences for the language composite score were −2.36 (95% CI, −12.60 to 7.88; *P* = .65) for the MT at NICU with postdischarge SC group, 2.65 (95% CI, −7.94 to 13.23; *P* = .62) for the SC at NICU and postdischarge MT group, and −3.77 (95% CI, −13.97 to 6.43; *P* = .47) for the MT group at both NICU and postdischarge. There were no significant effects for cognitive or motor development.

**Conclusions and Relevance:**

This secondary analysis did not confirm an effect of parent-led, infant-directed singing on neurodevelopment in preterm children at 24 months’ CA; wide CIs suggest, however, that potential effects cannot be excluded. Future research should determine the MT approaches, implementation time, and duration that are effective in targeting children at risk for neurodevelopmental impairments and introducing broader measurements for changes in brain development.

**Trial Registration:**

ClinicalTrials.gov Identifier: NCT03564184

## Key Points

**Question:**

What is the effect of parent-led, music therapist–supported, infant-directed singing, initiated during the neonatal intensive care unit stay and/or through 6 months after hospital discharge, on language development at 24 months’ corrected age?

**Findings:**

In this secondary analysis of a randomized clinical trial involving 112 children, receiving music therapy along with standard care up to 6 months of age led to language development at 24 months’ corrected age that was similar to development in children who received standard care only.

**Meaning:**

Findings of this study suggest that, although safe and well accepted by parents, parent-led, infant-directed singing compared with standard care did not result in greater language development benefits at 24 months’ corrected age in preterm children.

## Teaser

This secondary analysis of the LongSTEP randomized clinical trial compares cognitive, language, and motor scores at the 24-month follow-up among preterm infants receiving a music therapy intervention that included parent-led, infant-directed singing vs those receiving standard care.

## Introduction

Preterm birth is a major public health challenge worldwide affecting approximately 15 million neonates and their parents each year.^1^ Prematurity has a heavy burden on mortality, short-term severe morbidity, as well as long-term mental health and neurodevelopment of survivors.^2^ Although approximately 90% of preterm newborns survive without neurodevelopmental impairments,^2,3^ the brain is susceptible to dysfunction after preterm birth. Known neurodevelopmental impairments include poor cognitive and behavioral development^4,5^ and worse academic outcomes during childhood,^6,7^ posing challenges in adulthood.^7,8^

After preterm birth, the overwhelming auditory environment of the neonatal intensive care unit (NICU) may adversely influence plasticity of the auditory brain system, increasing the risk for language, attention, and cognitive deficits in preterm infants.^9,10^ Conversely, early exposure to adult speech in the NICU has been associated with better language and cognitive development at 7 and 18 months in preterm children.^11^ Parental voice benefits preterm infant vocalization,^12^ and infant-directed singing specifically captures infant attention and promotes expressive vocabulary and regulation.^13,14,15,16,17^ Meta-analyses have shown beneficial short-term effects of music therapy (MT) on infant heart rate, respiratory rate, oral feeding volume, and stress level.^18,19^ An increasing number of studies describe the role of MT in promoting beneficial neuroplasticity in preterm children,^20,21,22^ and recent evidence shows promising short-term neurophysiological outcomes.^23,24,25^ Three studies examined the effect of music interventions on neurodevelopment in preterm infants.^26,27,28^ Although all 3 studies suggested no effect on neurodevelopmental outcomes, 1 study demonstrated the benefit of early music listening for emotional outcomes at ages 12 and 24 months.^28^ All 3 studies used MT intervention delivered during NICU hospitalization. We could not identify any studies that used the Bayley Scales of Infant and Toddler Development, Third Edition (BSID-III) to examine the effect of MT delivered both during NICU hospitalization and after discharge from the hospital.

The Longitudinal Study of Music Therapy's Effectiveness for Premature Infants and Their Caregivers (LongSTEP) was a pragmatic randomized clinical trial (RCT) that evaluated the effects of MT through parent-led, infant-directed singing in the NICU and/or through 6 months after discharge.^29^ Previously reported results of this trial indicated no clinically important effects on primary (parent-infant bonding) or secondary (parental stress and mental health and child development) outcomes at 6 months’ and 12 months’ corrected age (CA).^30^ The current predefined secondary analysis evaluated longer-term effects on preplanned secondary outcomes: neurodevelopment at 24 months’ CA as measured by the BSID-III. The aim of this study was to evaluate the effect of the MT intervention during NICU stay and/or after hospital discharge on language development at 24 months’ CA. We predefined language development, as measured by the BSID-III language composite score, as the primary outcome; the remaining BSID-III composite and subscale scores were the secondary outcomes. We hypothesized that receiving music therapist–supported, parent-led, infant-directed singing would enable attuned musical interactions that promote mutual regulation and thereby facilitate neurodevelopment over time, leading to improved language, cognitive, and motor outcomes at 2 years of age.

## Methods

### Study Design and Participants

Children and their parents participated in the LongSTEP study, a 2 × 2 factorial, international, multicenter, assessor-blind pragmatic RCT conducted in 7 level-III and 1 level-IV NICUs across Argentina, Colombia, Israel, Norway, and Poland from August 2018 to April 2022. The original trial protocol is provided in Supplement 1. The intervention protocol, baseline characteristics of participants, and results from 2 independent feasibility studies have been published elsewhere.^29,31,32,33,34^ The Regional Committees for Medical and Health Research Ethics in Norway and the relevant ethics committees in each participating country approved the study. Written informed consent was obtained from parents or caregivers. We followed the Consolidated Standards of Reporting Trials (CONSORT) reporting guideline.

Between August 2018 and April 2020, we approached and recruited preterm infants and their parents during their NICU stay. Eligible infants were born before 35 weeks’ gestation, determined by medical staff to be sufficiently medically stable to start the intervention, and likely to stay at the NICU longer than 2 weeks from the time of recruitment. Eligible parents were those who could provide written, site-specific informed consent; were willing to actively participate in MT sessions; lived within reasonable commuting distance from the NICU to facilitate participation after discharge; and had sufficient understanding of the respective national language and mental capacity to complete the intervention and questionnaires.^29^

### Randomization

Eligible participants were enrolled and underwent baseline assessments. The LongSTEP trial’s factorial design included 2 phases: NICU hospitalization and follow-up after hospital discharge, with 2 randomization time points. At the NICU, participants were first randomized to MT plus standard care (SC) or SC alone. A computer-generated randomization list with a 1:1 ratio, in block sizes of 2 or 4 varying randomly, was used and stratified by site. The randomization sequence was generated by a researcher with no clinical involvement (C. Gold) and distributed using an online system (Sealed Envelope; Sealed Envelope Ltd). The allocation status was entered into a web-based system for electronic data capture and clinical data management for multicenter clinical trials (OpenClinica; OpenClinica, LLC). In cases of multiple pregnancies, we randomized and included only the first-born infant while all multiple-birth siblings received the same intervention. Before discharge, participants were randomized again to postdischarge MT or SC in a 1:1 ratio, using the same procedures.

### Procedures

#### MT Intervention

The approach to MT, parent-led, infant-directed singing, in the LongSTEP trial^29^ was individualized to each family and focused on mutual beneficial effects for both children and parents. The approach builds on MT theory^35,36,37,38^ and research^39,40^ integrated with concepts from developmental psychology and neuroscience.^33^ It is (1) resource oriented; (2) a musical dialogic process, with therapeutic intent aimed at the parent-infant dyad or triad; (3) culturally relevant; (4) focused on a high level of parent involvement to foster parental identity; (5) individualized; (6) progressively developmentally appropriate based on the infant’s emerging awareness and responsiveness over time; and (7) infant directed.^33^ Music was adapted to the parent’s culture, musical preferences, and parental abilities but was still guided by specific principles to ensure similar intervention for the included participants.^33,41^

The MT intervention consisted of 3 weekly individual sessions of approximately 30 minutes each (with a maximum of 27 sessions) during NICU hospitalization and 7 individual sessions of approximately 45 minutes each across the first 6 months after discharge to home.^29^ Interventions were provided by 11 music therapists with or in the final stage of completing a master’s degree in MT. These music therapists had clinical experience and training in the use of MT in neonatal contexts and received training on the protocol and supervision from the study core team to enhance protocol adherence. Treatment fidelity evaluations demonstrated that music therapists across the sites delivered the intervention with satisfactory adherence to the guiding principles.^41^

#### Standard Care

Standard care included early intervention methods of medical, nursing, and social services, with the exception of MT approaches. Families were asked not to engage in MT outside of the study context through the 12-month study period.^29^

### Outcomes and Blinding

This analysis used baseline and discharge data^32^ along with neurodevelopmental data gathered at 24 months’ CA. As mentioned, the BSID-III language composite score was set as the primary outcome, and the other composite and scaled scores were the secondary outcomes.

Neurodevelopment was assessed using the BSID-III when the children were approximately 2 years of age.^42^ The BSID-III, an individually administered instrument used worldwide for children up to 42 months of age, is considered the gold standard for evaluating early child development in clinical work and research^43^ and is used extensively with preterm infants.^44^ The BSID-III includes 3 assessor-evaluated developmental domains: cognitive, language, and motor, which are expressed in composite scores standardized with a population mean (SD) of 100 (15). The BSID-III also includes subscales (receptive and expressive language; fine and gross motor), which are expressed in scaled scores ranging from 1 to 19, with a population mean (SD) of 10 (3). Higher scores indicate better development. Two parent-reported BSID-III subscales on adaptive and socioemotional development were not included in the current study.

Assessments were completed by 11 qualified independent assessors (2 from Poland, 3 from Israel, 1 from Argentina, 1 from Colombia, and 4 from Norway) following the manual without local adjustments to the items. All, except for the Polish assessors, had prior experience with conducting BSID-III assessments. Prior to the study, assessors received individualized virtual training, including standardization procedures (eAppendix in Supplement 2), from senior psychologists (Ł.B., I.K.). Assessments were scheduled at 24 months’ CA (within a 2-week window). Parents were approached by the site investigator or assessors by post, email, and/or telephone or text messaging approximately 4 weeks before the infant reached 24 months’ CA to schedule an appointment within the target window period. Due to COVID-19–related challenges, the window period was extended to 32 months’ CA. Age was adjusted for prematurity for all, also for those older than 24 months at assessment,^45^ when converting raw scores into scaled and composite scores using the US norms. Assessments were completed at the hospital, clinic, research institution, or in exceptional cases the family’s home. Regardless of location, all aspects of the environment were controlled as much as possible in order to obtain a reliable assessment of the child’s development. Parents or caregivers were present during the assessment. When assessments were completed, the assessor provided feedback and recommendations to parents according to local standards, ensuring appropriateness to the participant’s culture.

Assessments were performed by assessors who were blind to participants’ group randomization. After assessments were completed, assessors were asked whether they inadvertently had become aware of the randomization. Success of blinding was verified in all assessments.

### Statistical Analysis

The target sample size was calculated for the main outcome of the LongSTEP trial (parent-infant bonding),^29^ with 80% power for a medium effect size (Cohen *d* = 0.5) and 20% attrition in a 2-sided test with 5% significance level. Therefore, the present study would also be able to detect medium-sized effects (corresponding with a mean [SD] score of 7.5 [15] points) on the BSID-III.

Descriptive statistics for participants with data at follow-up (mean [SD]; No. [%]) were generated for all variables included in this study. Treatment effects were investigated using linear mixed-effects models (LMEs) with site as a random effect. All tests were 2-sided with a *P* < .05 significance level. LMEs make optimal use of all available data, providing the basis for the main intention-to-treat analysis. Sensitivity intention-to-treat analysis was based on multiply imputed complete datasets. Post hoc, we explored clinical and demographic factors of language composite scores and associated intervention effects in LMEs. We conducted analyses using R, version 4.3.0 (R Project for Statistical Computing).

## Results

**Figure 1.**
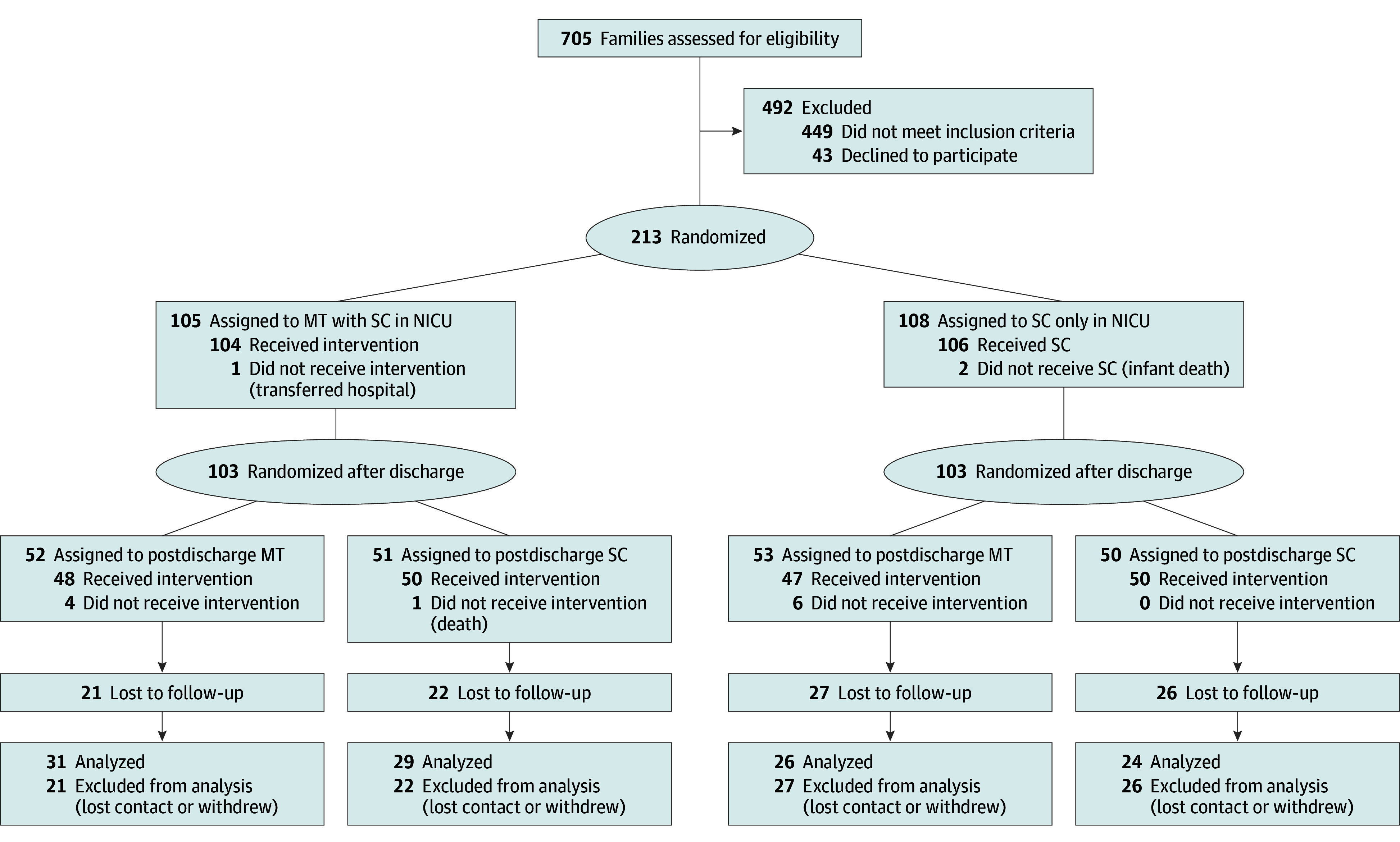
Study Flow Diagram of Families in the LongSTEP Trial MT indicates music therapy; NICU, neonatal intensive care unit; SC, standard care.

A total of 206 families consented to participate and were randomized to 4 intervention groups at enrollment (51 to MT with SC; 53 to SC at NICU) and discharge (52 to MT; 50 to SC). Of the original sample (103 females [50%], 103 males [50%]; mean [SD] gestational age, 30.5 [2.7] weeks; mean [SD] birth weight, 1400.5 [432.8] grams), 112 (54%) were included in the follow-up at 24 months’ CA (mean [SD] CA at assessment, 25.54 [1.72] months) (Figure 1). Baseline characteristics of children and parents were well balanced between the intervention groups (Table 1)^46^; we found no difference between those who were followed up and those who were lost to follow-up (eTable 1 in Supplement 2). Retention rates varied across countries but not by intervention group; 1 site had high retention (12 of 54 [22%]), which shows that it is possible to maintain contact with families over a long period (eTable 2 in Supplement 2). Intervention statistics and adverse event data are published elsewhere.^30^

**Table 1.** Baseline Characteristics Across 4 Intervention Groups^a^

Characteristic	SC group		MT at NICU with postdischarge SC group		SC at NICU and postdischarge MT group		MT at both NICU and postdischarge group	
	Total No.	No. (%)	Total No.	No. (%)	Total No.	No. (%)	Total No.	No. (%)
Child sex								
Female	50	25 (50)	51	23 (45)	53	30 (57)	52	25 (48)
Male	50	25 (50)	51	28 (55)	53	23 (43)	52	27 (52)
Singleton pregnancy	50	33 (66)	51	38 (75)	53	43 (81)	52	31 (60)
Cesarean delivery route	50	43 (86)	51	39 (76)	53	44 (83)	52	42 (81)
Birth weight, mean (SD), g	50	1422.86 (428.20)	51	1364.04 (426.30)	52	1424.02 (464.08)	52	1391.27 (420.94)
GA at birth, mean (SD), wk	50	30.89 (2.61)	51	30.37 (2.59)	53	30.48 (2.79)	52	30.18 (2.61)
<28		6 (12)		9 (18)		9 (17)		12 (23)
28-32		23 (46)		23 (45)		19 (36)		22 (42)
32 to <35		21 (42)		19 (37)		25 (47)		18 (35)
PMA enrollment, mean (SD), wk	50	33.11 (1.69)	51	33.32 (2.34)	53	33.1 (1.69)	52	32.77 (2.06)
Apgar score at 5 min, mean (SD)	49	8.53 (1.43)	50	8.42 (1.67)	53	8.68 (1.09)	49	8.8 (1.14)
Weight enrollment, mean (SD), g	48	1582.25 (390.92)	51	1675.49 (494.89)	52	1640.48 (372.01)	52	1577.38 (406.50)
Estimated severity of IVH^b^								
Cranial ultrasonography not indicated	50	22 (44)	51	17 (33)	52	21 (40)	52	27 (52)
None		25 (50)		27 (53)		24 (46)		21 (40)
Grade 1-2		3 (6)		5 (10)		6 (12)		4 (8)
Grade 3-4		0		2 (4)		1 (2)		0
Maternal age, mean (SD), y	50	33.4 (5.58)	51	32.14 (5.15)	51	32.22 (5.71)	51	34.08 (5.34)
Maternal educational level, mean (SD), y	48	15.56 (2.62)	50	15.08 (3.85)	50	15.9 (2.77)	51	16.49 (2.63)
Maternal civil status^c^								
Married or living with partner	50	45 (90)	50	47 (94)	53	47 (89)	52	49 (94)
Maternal work status^d^								
Full-time or self-employed	50	39 (78)	51	34 (67)	53	40 (75)	52	37 (71)
Paternal age, mean (SD), y	47	36.57 (7.14)	48	35.17 (6.73)	50	34.48 (5.79)	49	36.53 (5.08)
Paternal educational level, mean (SD), y	44	14.98 (3.11)	47	14.3 (3.42)	49	15.29 (3.42)	47	15.64 (3.23)
Paternal work status^d^								
Full-time or self-employed	47	43 (91)	48	44 (92)	50	49 (98)	49	44 (90)

Abbreviations: GA, gestational age; IVH, intraventricular hemorrhage; MT, music therapy; NICU, neonatal intensive care unit; PMA, postmenstrual age; SC, standard care.

^a^ For 206 participants who were randomized twice.

^b^ IVH was diagnosed by cranial ultrasound and graded according to Papile et al.^46^

^c^ Same-sex parents were invited to participate in the study, but none were enrolled.

^d^ Other status included part-time, homemaker or stay-at-home parent, and student.

**Figure 2.**
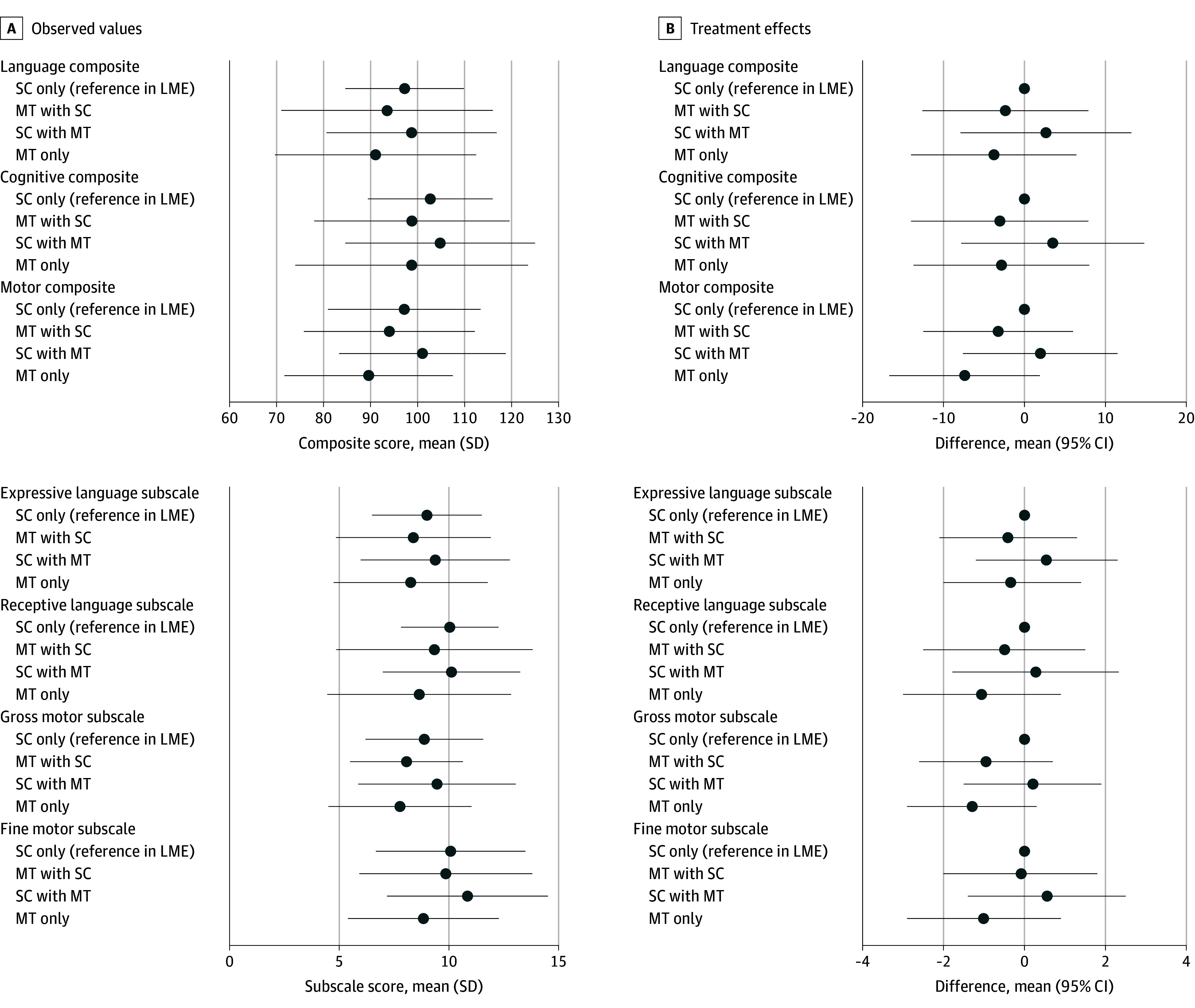
Mean Bayley Scales of Infant and Toddler Development, Third Edition Composite and Subscale Scores and Treatment Effects in the 4 Intervention Groups LME indicates linear mixed-effects models; MT, music therapy; and SC, standard care. Circles represent mean composite and subscale scores and mean differences, and whiskers represent SDs for the composite and subscale scores and 95% CIs for the mean differences.

Observed values of the BSID-III composite and subscale scores in the 4 intervention groups were similar (Table 2; Figure 2). Across groups, the mean (SD) language composite score was 94.7 (19.3), with a range from 53 to 135. The mean (SD) for the cognitive composite score was 100.8 (20.6), with a range from 55 to 145, and the mean (SD) for the motor composite score was 95.0 (17.9), with a range from 46 to 154. Most participants (79 [70%] to 93 [83%]) had BSID-III scores in the normal range (≥85). In the overall sample, 33 infants (30%) demonstrated language composite scores below the normal range (<85), while 19 (17%) and 29 (26%) had cognitive and motor scores below the normal range. Mean differences for the language composite score were −2.36 (95% CI, −12.60 to 7.88; *P* = .65) for the MT at NICU with postdischarge SC group, 2.65 (95% CI, −7.94 to 13.23; *P* = .62) for the SC at NICU and postdischarge MT group, and −3.77 (95% CI, −13.97 to 6.43; *P* = .47) for the MT group at both NICU and postdischarge, all compared with the SC group (Table 2). The mean differences for secondary outcomes were all not significant (Table 2; Figure 2).

**Table 2.** Mean Bayley Scales of Infant and Toddler Development, Third Edition (BSID-III) Composite and Subscale Scores in the 4 Intervention Groups and Mean Differences

BSID-III domains	SC group (n = 24) [Reference]	MT at NICU with postdischarge SC group (n = 29)			SC at NICU and postdischarge MT group (n = 26)			MT at both NICU and postdischarge group (n = 31)			No. of participants
	Mean scores (SD)	Mean scores (SD)	Mean difference (95% CI)^a^	*P* value	Mean scores (SD)	Mean difference (95% CI)	*P* value	Mean scores (SD)	Mean difference (95% CI)	*P* value	
Language composite	97.25 (12.61)	93.52 (22.50)	−2.36 (−12.60 to 7.88)	.65	98.73 (18.10)	2.65 (−7.94 to 13.23)	.62	91.06 (21.41)	−3.77 (−13.97 to 6.43)	.47	110
Expressive subscale	9.00 (2.50)	8.38 (3.53)	−0.41 (−2.13 to 1.31)	.64	9.38 (3.40)	0.54 (−1.24 to 2.31)	.55	8.26 (3.51)	−0.34 (−2.05 to 1.37)	.69	110
Receptive subscale	10.04 (2.22)	9.34 (4.48)	−0.49 (−2.48 to 1.50)	.63	10.12 (3.13)	0.28 (−1.78 to 2.33)	.79	8.65 (4.19)	−1.06 (−3.03 to 0.92)	.29	110
Cognitive composite	102.71 (13.27)	98.79 (20.77)	−3.04 (−14.07 to 7.57)	.58	104.81 (20.17)	3.49 (−7.89 to 14.47)	.54	98.75 (24.76)	−2.84 (−13.85 to 7.56)	.60	111
Motor composite	97.17 (16.24)	94 (18.18)	−3.25 (−12.54 to 6.04)	.49	101.04 (17.72)	1.97 (−7.56 to 11.50)	.68	89.60 (17.91)	−7.38 (−16.66 to 1.88)	.12	108
Gross motor subscale	8.88 (2.68)	8.07 (2.57)	−0.95 (−2.58 to 0.69)	.25	9.46 (3.59)	0.21 (−1.46 to 1.89)	.80	7.77 (3.26)	−1.29 (−2.92 to 0.34)	.12	108
Fine motor subscale	10.08 (3.41)	9.86 (3.94)	−0.08 (−2.00 to 1.85)	.94	10.85 (3.67)	0.56 (−1.41 to 2.53)	.57	8.84 (3.44)	−1.01 (−2.92 to 0.90)	.29	109

Abbreviations: MT, music therapy; NICU, neonatal intensive care unit; SC, standard care.

^a^ Results from linear mixed-effects models with site as random effect.

Male sex and oxygen supplementation were risk factors for lower language composite scores (eTable 3 and eFigure 1 in Supplement 2) but were not associated with intervention effects (eTable 4 and eFigure 2 in Supplement 2). Sensitivity analyses using multiple imputation (eTable 5 in Supplement 2) did not show significant effects.

## Discussion

Results suggest that children who received MT at NICU with postdischarge SC demonstrated similar developmental scores as children who received SC only. This finding means that we could not confirm a beneficial or detrimental MT effect on neurodevelopment in these premature children. Wide CIs suggest, however, that meaningful effects in either direction cannot be excluded.

Although we hypothesized that MT has an effect on language development at 24 months’ CA, the lack of favorable findings is consistent with recent studies demonstrating no clinically meaningful effects of creative MT on the BSID-III in 82 preterm children at 24 months’ CA^26^ and no effect of parent-led, infant-directed singing supported by a music therapist on BSID-III measured at age 2 to 3 years.^27^ Both the current study and recent studies used individually tailored MT during NICU hospitalization, emphasizing the communicative capacity of the infant. The results are also similar to those of a study of music-listening intervention during NICU hospitalization not involving a music therapist,^28^ demonstrating no effect on the BSID-III cognitive, language, and motor domains in preterm children at 12 months. Hence, in terms of effects of music-based interventions provided during NICU hospitalization on BSID-III scores at 24 months’ CA in preterm children, the findings of this analysis confirmed previous null findings (eFigure 3 in Supplement 2).^26,28^ Notably, in all studies, including the present work, mean BSID-III scores were within the normal range, questioning the need for additional intervention in the study populations. An asset of this study is the ability to test the potential effects of MT in the NICU and/or MT after discharge compared with SC. Estimates suggest no difference in outcomes across condition. To our knowledge, no study has examined the effect of postdischarge MT on BSID-III scores in preterm infants, and we were unable to compare the current findings with previous results.

Although we hypothesized that music therapist–supported, parent-led, infant-directed singing would support infant neurodevelopment over time, we were not able to prove such an effect in the current study. There could be several possible explanations for the null findings. First, previous reports suggested that infants in the sample were medically stable and mature at enrollment,^41^ which was underscored by a large proportion of children having BSID-III scores within the normal range at the 24-month follow-up. Hence, this sample was not the group of premature infants most vulnerable to neurodevelopmental impairment, which may have limited the opportunity for intervention effects. Second, the instability of early child development measures^47^ may lead to challenges in identifying group differences. This explanation is of particular importance in the case of some but not all preterm children with an increased risk of delayed development in the first years of life.^48^ In the current study, the wide CIs of the effect estimates and comparably larger SDs in the intervention groups could indicate large variability of the outcomes, suggesting that some but not all infants benefitted from the MT intervention. Moreover, although the BSID-III is considered a gold standard instrument in early childhood, it might not be sufficiently sensitive to capture the hypothesized subtle changes in the brain from MT.^49,50^ Thus, follow-up in later childhood using tools with higher sensitivity and specificity (eg, Wechsler Intelligence Scale for Children, Fifth Edition; Stanford-Binet Intelligence Scales, Fifth Edition) may yield different findings. Alternatively, other procedures, such as heart rate variability, electroencephalogram, or functional near-infrared spectroscopy, could be more suited to identify the effect of MT on brain functioning.^16,17,51^ Future studies should focus on identifying those infants who could benefit from early MT and measuring the effects with a wider array of neurodevelopmental and neurophysiological instruments.

### Strengths and Limitations

Key strengths of the LongSTEP trial were the pragmatic approach in setting (a diversity of clinical settings) and the intervention completion, increasing the generalizability of the findings. The pragmatic approach, however, could also represent a limitation, introducing noise in the data. The onset of the COVID-19 pandemic and lockdowns aggravated this risk in several ways. Retaining families to participate in follow-up is already challenging,^52^ but it is even more so during the COVID-19 pandemic, leading to a low study retention rate (54%) and, as a consequence, underpowered analyses for detecting the effect of MT on neurodevelopmental outcomes.^53,54^ Retention differed by participating country, probably due to differences in clinical practice. Notably, 1 site had high retention (12 of 54 = 22% attrition), showing that it is possible in principle to keep contact with families over a longer time span. Moreover, pandemic-related lockdowns affected the quality of the BSID-III training, standardization, and quality control process, impairing the reliability of the measures. The onset of the pandemic also extended the assessment window from 24 to 32 months’ CA (within a 2-week window). However, although 53 children were assessed outside the window period, the mean (SD) CA at assessment was 25.54 (1.72) months, suggesting minimal deviation from the original age window. Furthermore, although testing was planned to be administered in designated rooms at the clinic or hospital, some assessments were also performed at the family’s home due to lockdowns. Noise introduced by the COVID-19 pandemic and high attrition rate increased the risk of type II error and the risk of our inability to detect effects that were actually present.

## Conclusions

In this secondary analysis of a pragmatic RCT, a music therapist–supported, parent-led, infant-directed singing intervention did not show effects on neurodevelopment at 24 months’ CA. Wide CIs suggest that meaningful effects in either direction cannot be excluded. Future research with a focused RCT design is warranted to determine the MT approaches, implementation time, and duration that are effective in targeting groups at risk for neurodevelopmental impairment and introducing broader measurements for changes in brain development.

## Notes

Supplement 1.
Trial Protocol

Supplement 2. **eAppendix.** Standardization Procedures of Neurodevelopmental Assessment With the Bayley Scales of Infant and Toddler Development, 3rd Edition (BISD-III) **eTable 1.** Infant Baseline Characteristics of Those Followed-Up Versus Lost to Follow-Up **eTable 2.** Completers by Country and Intervention Group **eTable 3.** Clinical and Demographic Predictors of the Bayley Scales of Infant and Toddler Development, 3rd Edition (BISD-III) Language Composite Score **eFigure 1.** Estimates of Clinical and Demographic Predictors of the Bayley Scales of Infant and Toddler Development, 3rd Edition (BISD-III) Language Composite Score **eTable 4.** Intervention Effects by Selected Clinical/Demographic Predictors of the Bayley Scales of Infant and Toddler Development, 3rd Edition (BISD-III) Language Composite Score **eTable 5.** Sensitivity Analysis: Estimates of Intervention Effects on the Bayley Scales of Infant and Toddler Development, 3rd Edition (BISD-III) Language Composite Score Using Multiple Imputation **eFigure 2.** Estimates of Intervention Effects on the Bayley Scales of Infant and Toddler Development, 3rd Edition (BISD-III) Language Composite Score by Selected Clinical/Demographic Predictors **eFigure 3.** Effects of Music Interventions on the Bayley Scales of Infant and Toddler Development, 3rd Edition (BISD-III) at 24 Months Across Studies
eReferences

Supplement 3.
Data Sharing Statement
