# Errors in the Strength and Limitations and Supplement 2

**DOI:** 10.1001/jamanetworkopen.2024.26477

**Published:** 2024-07-12

In the Original Investigation “Music Therapy in Infancy and Neurodevelopmental Outcomes in Preterm Children: A Secondary Analysis of the LongSTEP Randomized Clinical Trial”^1^ published on May 16, 2024, there were errors in the Strengths and Limitations section and Supplement 2. “Attrition” was omitted after a percentage in the Strengths and Limitations section. The sentence should be as follows: “Notably, 1 site had high retention (12 of 54 = 22% attrition), showing that it is possible in principle to keep contact with families over a longer time span.” Additionally, there was a typographical error in the eFigure 3 caption in Supplement 2. This article has been corrected.
