# Supplementary material for: Music Therapy in Infancy and Neurodevelopmental Outcomes in Preterm Children: A Secondary Analysis of the LongSTEP Randomized Clinical Trial
Source: JAMA Netw Open. 2024 May 16;7(5):e2410721. doi: 10.1001/jamanetworkopen.2024.10721 (PMC11099691; doi:10.1001/jamanetworkopen.2024.10721)
Supplement: Supplement 1. — Trial Protocol [file jamanetwopen-e2410721-s001.pdf]

Longitudinal Study of music Therapy's Effectiveness for Premature infants and their caregivers: international randomized trial (LongSTEP) (Forskerprosjekt - BEHANDLING)

Application Number: ES592623 Project Number: -1

Applicant

Project Owner

Institution / company (Norwegian name)	Uni Research AS
Faculty	
Institute	
Department	Uni Research Health
Address	Postboks 7810
Postal code	5020
City	Bergen
Country	Norway
E-mail	VVVV
Website	uni.no/helse
Enterprise number	VVVV

eAdministration	

Project administrator

First name	IVVVV
Last name	VVVV
Date of birth	VVVV
Personal number	
Gender	VVVV
Position/title	Research Director
Phone	VVVV
E-mail	helse@uni.no
Confirmation	✔ The application has been approved by the

	Project Owner

Project manager

First name	Christian
Last name	Gold
Date of birth	VVVV
Personal number	VVVV
Gender	VVVV
Institution / company (Norwegian name)	Uni Research AS
Faculty	
Institute	
Department	Uni Research Health
Address	Postboks 7810
Postal code	5020
City	Bergen
Country	Norway
Position/title	Principal researcher
Academic degree	PhD
Preferred language	Bokmål
Phone	VVVV
E-mail	VVVV

Project info

Project title

Project title	Longitudinal Study of music Therapy's Effectiveness for Premature infants and their caregivers: international randomized trial (LongSTEP)

Primary and secondary objectives of the project

Primary and secondary objectives	Primary objective:

	1. To provide high-quality evidence of the effects of music therapy on bonding between preterm infants and their parents during neonatal hospitalization and in primary care post-discharge.<br><br>Secondary objectives:<br>2. To examine effects of music therapy on premature infants' general and socio-emotional development and social behaviour.<br>3. To examine effects of music therapy on premature parents' depression and stress.<br>4. To examine medical and social factors of premature infants and their parents that may be associated with music therapy, including use of services.<br>5. To prepare for examining cost-effectiveness and longer-term evaluation of effects beyond the first year of life.

Project summary

Project summary	Background: Preterm birth is a major medical, psychological and socio-economic problem worldwide. Music therapy (MT) is part of best practice in neonatal care in some countries, but is only emerging in Norway. A recent systematic review suggests positive effects of MT for preterm infants and parents/caregivers, but methodologically rigorous studies including long-term follow-up of infant and parental outcomes are missing.<br><br>Objectives: Our aim is to provide evidence of the impact of MT on infant/caregiver bond, caregiver well-being and infant development. We will examine the effectiveness and feasibility of implementing MT within the Norwegian, European and international context, bridging specialist care and municipal health care settings. We will also provide the basis for examining cost-effectiveness and long-term outcomes beyond the project period.<br><br>Methods: The study is a 2x2 factorial, international multi-center, assessor-blind pragmatic randomized controlled trial evaluating the longitudinal effect of MT on preterm infants and primary caregivers across 12 months. MT will be delivered during NICU and/or during a post-discharge 6-month period. The Postpartum Bonding Questionnaire will be used to evaluate bond. Parental depression will be assessed with Edinburgh Postnatal Depression Scale, and parental stress with Parental Stress Scale. Other standardized scales will be used to assess infant psycho- and socio-emotional development.<br><br>Discussion: This study fills a gap in knowledge by assessing the longer-term impact of MT for preterm infants/caregivers, and of MT beyond the hospital context. Study outcomes are directly applicable to the development of clinical practice and will inform future research, theory and practice on a national and international level. By incorporating family-centered care, continuity of care,

	user involvement, and cultural relevance, this study contributes to improved rehabilitation.

Funding scheme

Supplementary info from applicant

Programme / activity	BEHANDLING
Application type	Forskerprosjekt
Topics	
Other relevant programmes/ activities/projects	
Discipline(s)	Psychology, music therapy, pediatrics
If applying for additional funding, specify project number	
Have any related applications been submitted to the Research Council and/or any other public funding scheme	Yes
If yes, please provide further information	We submitted a similar project last year, which was rejected then, but it has been revised substantially.

Progress plan

Project period

From date	20180101
To date	20211231

Longitudinal Study of music Therapy's Effectiveness for Premature infants and their caregivers: international randomized trial (LongSTEP)

1. Relevance

The proposed project is **an international multicenter pragmatic randomized controlled trial (RCT)** assessing the longitudinal impact of music therapy's (MT's) effects on a **clinically relevant primary endpoint** in premature infants and their parents. Preterm birth is "one of the **largest single conditions in the Global Burden of Disease** analysis" [1], with high mortality and risk of lifelong impairment. This trial will determine whether MT as delivered by a qualified music therapist during neonatal intensive care unit (NICU) hospitalization and/or in community care after discharge to home is superior to standard care in improving **bonding between** primary caregivers and preterm infants, an outcome that is linked to **parental well-being and infant development. Building on** promising results from **phase II studies and meta-analyses**, LongSTEP is the **first phase III trial** worldwide of MT's effectiveness for premature infants and their caregivers. This project promotes **continuity of care** by **bridging specialist and municipal health care** contexts, and uses **family-centered developmental care** to treat the infant within the family context. This protocol links **two health regions** in Norway with international clinical sites in **five countries** (Norway, Sweden, Germany, Israel, and Columbia). **Outcomes** are **clinically relevant** and use **electronic health registry data** where this is possible. It features **interdisciplinary collaboration** between neonatologists, psychologists, music therapists, trial methodologists, biostatisticians, and health economists; preparations towards a full **cost-effectiveness** analysis are included. **User representatives** have confirmed the project's importance and relevance and will continue to be **involved in all aspects** from planning and conducting the trial to disseminating and implementing its findings. LongSTEP has strong potential for publication in high-ranked international journals and will contribute to future practice development, theory, and research. Findings will be **directly applicable to clinical practice**, and will have a valuable impact for infants from an at-risk group, their primary caregivers, and the practitioners working with them. MT is relevant for both **genders** and can be tailored to **minorities** with different cultural backgrounds.

2. Aspects relating to the research project

2.1 Background and status of knowledge

The global average preterm birth rate is around 11% worldwide corresponding to 15 million preterm infants each year [1, 2]. Over one million die as a direct result of prematurity and another million show lifetime impairment [3]. With advancements in medical technology, a growing number of neonates as young as 23 to 24 weeks gestational age (GA) with birth weight as low as 500 grams are surviving in high-income countries. In Norway, 3275 children (5.5%) were born at 28-36 weeks GA, and 230 (0.5%) at 22-27 weeks GA in 2012 [4]. Preterm infants have higher risks of mortality, morbidity, and various postnatal complications. With improved survival rates, there is growing concern for long lasting complications including behavioral and mental health problems, and poorer quality of life [1]. Moreover, the economic burden of preterm birth includes increased cost of neonatal care, long-term costs associated with complex health status, and lost economic productivity over life [5].

Prematurity strongly impacts caregivers. Premature labor is perceived as a stressful [6], traumatic [7, 8], unexpected event that interrupts mothers' antenatal bonding and preparation for delivery. Families can experience higher levels of stress, anxiety, and fear for the infant's safety, as well as insecurity and powerlessness [8, 9]. Mothers are at risk for postpartum depression, posttraumatic stress disorder, feelings of guilt, mourning, and lack of self-worth. Separation due to NICU stay and prolonged hospitalization can complicate the development of healthy infant/parent bonding and adversely impact the formation of secure attachment [8, 10]. Preterm birth and subsequent hospitalization can impair the mother-infant relationship, which plays a central role in the child's socio-emotional development and formation of future intimate relationships [11, 12]. Mothers' psychological well-being [13] or postpartum depression [14] are the most important factors affecting infant/parent relationship in the postnatal period. Decreasing maternal stress and

reducing early separation during NICU hospitalization are crucial aims for positively impacting long-term outcomes [15].

MT is part of NICU standard treatment in some hospitals in several countries. MT in infant critical care involves the informed use of music and a therapeutic relationship to promote infant development and facilitate secure attachment with primary caregivers [16]. MT promotes infant sensory regulation and encourages neurological development [17, 18]. Incorporating parents as active partners in implementing NICU-MT decreases infant and parent distress [19], which may promote bonding. MT beneficially impacts infant physiological parameters, behavior state, weight gain and feeding ability, and may reduce hospital stay [17, 20-24]. In a rigorous, updated systematic review and meta-analysis of RCTs we confirmed a large, favorable effect of MT on respiratory rate and maternal anxiety [25]; however, several areas require further investigation. Rigorously designed and adequately powered studies using standardized outcome measures and interventions implemented by music therapists with specialized NICU training are required. Parallel RCTs to evaluate long-term effects of MT, and extending intervention periods past discharge from NICU are required to assess long-term impact, a substantial gap in present knowledge [25]. Studies examining longitudinal provision of MT tailored to different stages of infant development [19] and long-term effects of MT on the development of healthy infant/parent bonding and secure attachment are needed [17, 25].
Objectives: see grant application form.

2.2 Approaches, hypotheses and choice of method

2.2.1 Study design

We propose a factorial (2x2), multi-center, assessor-blind pragmatic RCT to evaluate the longitudinal effect of MT in NICU and/or after discharge on premature infants and their primary caregivers across a 12-month time period (Fig. 1). The factorial design will allow testing the independent effect of each combination: NICU-MT, post-discharge MT, both, or neither, as well as interactions between them. Participants will initially be recruited from the NICU of participating hospitals according to the following in- and exclusion criteria:

(1) Infants: Born below 35 weeks GA, of both genders, any ethnicity, from single or multiple pregnancies, who have achieved sufficient medical stability (as determined by medical staff) to start MT.
(2) Primary caregivers: One of the parents/caregivers must provide written, informed consent before any study procedures occur. Primary caregivers should agree to engage in at least 2 of the 3 MT sessions per week during NICU, and/or in 5 of the 7 MT post-discharge sessions. To ensure feasibility, primary caregivers should live within reasonable commuting distance from the treating NICU in order to be eligible for participation.

The 2x2 factorial design entails a two-phase protocol covering both the stay in the NICU and municipal care after discharge from hospital. During NICU stay, participants will be randomly allocated to receive either MT integrated in standard NICU care or standard NICU care alone. Before discharge to home, participants will again be randomly allocated to either MT integrated in municipal care or standard municipal care. Standard care may include other early intervention methods of care with the exception of other music-based approaches. Treatment fidelity (adherence and competence) will be evaluated by independent raters using video recordings.

2.2.2 Interventions

The MT approach in this study is informed by models previously described and tested [18, 21, 25, 26]. Like other MT models [26], it is based on the notion that infants have a capacity to communicate actively [27, 28]. As in some newer models, parents are actively involved and empowered [21, 29]. The approach here thus builds on an emerging consensus, but is more explicit in its adaption to developmental levels and emphasis on infant/parent co-regulation. Furthermore, unlike previous studies [25], MT is extended beyond the tertiary care setting to help with the transition to daily life and municipal care. MT focuses on supporting positive relationship formation within the infant/parent dyad by promoting beneficial co-regulation, and includes psychosocial support of the parent.

MT during NICU: Participants will be offered individual MT sessions 3 times/week for 20-30 minutes/session, with a maximum of 36 sessions per infant (assuming a maximum NICU stay of 9 weeks). NICU-MT will be provided for all eligible infants once they achieve medically stability, usually after 26 weeks postmenstrual age (PMA; defined as GA at birth plus the time since birth [30]). A flexible protocol of procedures matched to infant PMA, familial/cultural preferences, and infant's demonstrated readiness to receive stimulation, will occur as individually-tailored sessions at bedside:

Approximately 26 to 31.99 weeks PMA – Cautious use of sung/toned voice: The music therapist shows caregivers how to use facilitated tucking and midline alignment (if appropriate for the infant) along with

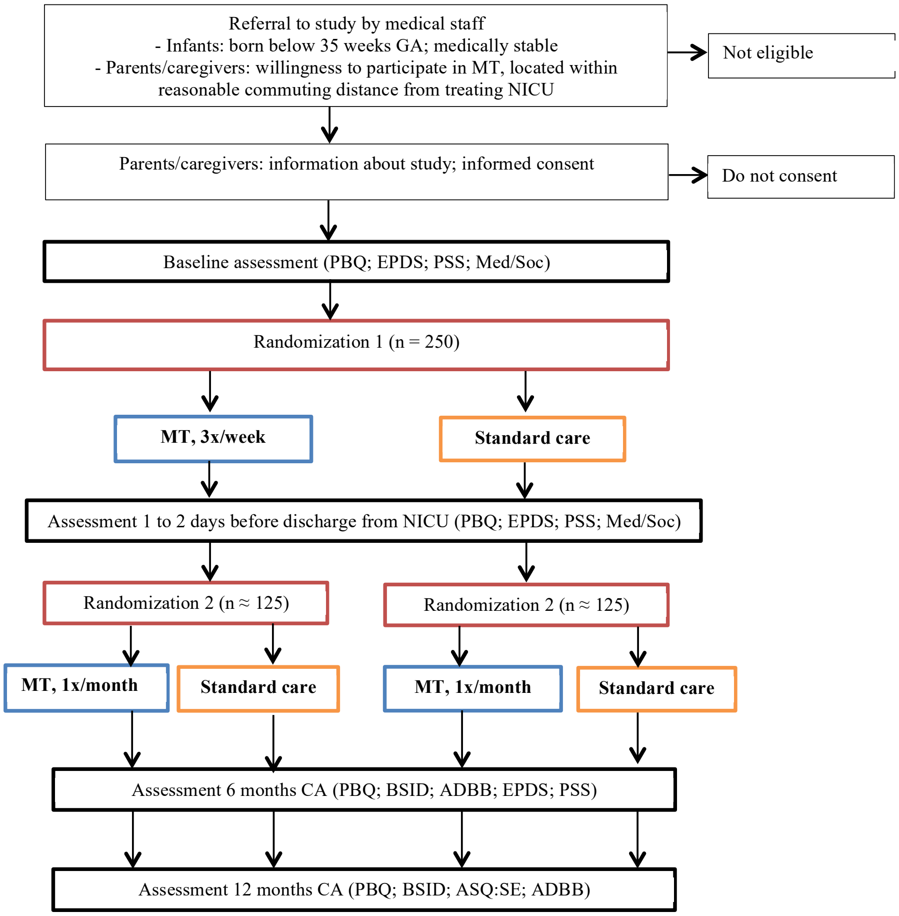

Figure 1. Flow-chart illustrating the design
Note. ADBB –Alarm Distress Baby Scale; ASQ:SE – Ages and Stages Questionnaire: Social-Emotional; BSID – Bayley Scales of Infant and Toddler Development; CA – corrected age; EPDS – Edinburgh Postnatal Depression Scale; Med/Soc – medical and social factors as detailed in text; PBQ – Postpartum Bonding Questionnaire; PSS – Parental Stress Scale.

sung or toned voice to pacify the infant, promote state regulation, and enable bonding, by using simple melodies or modifying songs of kin [21] into a lullaby style matched to infant responses. Use of sung/toned voice is adjusted in response to infant engagement/disengagement cues. The infant may be resting in his/her isolette with the portal door open, resting swaddled in his/her basinet or bed, or swaddled and cradled in a static manner by the caregiver (consistent with Kangaroo care) or the therapist. MT may occur during Kangaroo care, if Kangaroo care is part of standard care.
32 to 35.99 weeks PMA – Cautious use of multimodal stimulation: This phase applies as soon as the infant demonstrates readiness to receive additional sensory stimulation. The therapist reviews infant engagement/disengagement cues with the caregiver, describes techniques used in multimodal stimulation [18], models the progressive sequence, and supports caregivers in implementing it themselves: (1) Swaddling

and cradling infant statically (without tactile or vestibular stimulation at first). (2) Commencing contingent singing/humming of simple melodies or modified songs of kin while cradling infant. (3) Adding gentle massage while singing and cradling infant (delivered in cephalocaudal and proximodistal order [18]). (4) Gently rocking while providing massage, singing/humming, and cradling. (5) If the infant has begun opening his/her eyes, using vocal inflection to promote eye opening, eye contact, and responses to social cues (e.g., smiling, cooing). The sequence will be paced and modified according to infant engagement/disengagement cues, and will be reversed or paused in response to disengagement cues.
From 36 weeks PMA – Engagement in musical exchange: This phase applies when the infant demonstrates readiness to engage in more interactive levels of musical exchange. Caregivers interact musically with the infant, using vocal inflection and sung cues to encourage the infant to achieve a quiet, alert state and engage in eye contact, vocalization, and rudimentary social interaction. Depending upon infant readiness, caregivers may use premature infant-friendly, basic musical toys to promote auditory localization, auditory tracking, visual tracking, eye contact, reaching, grasping, and mouthing. The therapist will encourage the caregiver to use preferred lullabies and children's songs or to modify songs of kin [21] in order to encourage musical dialogue with the infant.
Standard care during NICU: This will vary across countries, but will typically include necessary medical care and a limited amount of interventions to reduce stress among infants and to inform and promote safety in parents [31].
MT post-discharge from hospital: Individual MT sessions will be offered, minimum 5 and maximum 7, 45-minute sessions per infant/parent distributed across six months. These will occur at municipal child health centers or at home, and comprise: (1) Verbal greeting and brief discussion of infant's progress (approx. 5 min). (2) Musical greeting of infant and caregiver, encouraging caregiver to participate (5 min). (3) Engagement in musical exchange following the procedures described in the "from 36 weeks" category above, with therapist modelling musical engagement (approx. 10 min). (4) Discussion of current infant/parent challenges and strategies for using musical interactions to address these needs, with therapist modelling musical techniques to promote self-regulation or to facilitate musical interaction, depending upon needs identified by the caregiver (approx. 10 min). (5) Caregiver demonstration of techniques discussed during session (approx. 10 min). (6) Musical closure and reminder of planned timing for next visit (approx. 5 min). Any siblings may be involved if desired by the caregiver. Subsequent sessions will follow a similar sequence, adjusted to infant developmental level and ongoing needs. The therapist will work in close dialogue with those providing standard aftercare procedures.
Standard care post-discharge from hospital: This includes follow-up visits and preventive interventions in primary or specialist health care as needed. Preventive interventions during the first year of life are focused on growth/eating/nourishment as well as psychomotor/sensory development and also include a focus on families and bonding [31]. According to our user representative (see separate document), support on psychological issues may however be limited and sometimes insufficient or inadequate.

2.2.3 Outcomes

Outcomes will be assessed at several time points: at baseline; one to two days before discharge to home; at 6 months corrected age (end of MT; primary endpoint); and 12 months of corrected age (CA; chronological age reduced by number of weeks born preterm [30]). Data collectors and assessors will be trained in assessment procedures and blinded to participant allocation; success of blinding will be verified.
Primary outcome: Bond between primary caregiver and infant will be evaluated at all time points ***(primary time point: 6 months CA)*** using the Postpartum Bonding Questionnaire (PBQ). PBQ is a self-rating screening instrument for disorders of the early mother-infant relationship consisting of 25 statements on a six-point Likert scale (each 0-5; sum score ranging from 0 to 125; high = problematic), addressing problems in the mother-infant relationship based on weakened bonding; rejection and anger; anxiety about care; and risk of abuse [32]. The scale is validated and widely used in clinical practice and research [33]. It has been translated and tested in several languages and cultures [34] and has high internal consistency [35] and test-retest reliability [32], also in the Norwegian version [36].
Secondary outcomes:
(1) Infant *development* will be assessed blindly using the Bayley Scales of Infant and Toddler Development (BSID). It covers cognitive, language, and motor development and is considered the gold standard for assessing development of young and premature infants up to 42 months [37].
(2) *Re-hospitalization* during the first year of life (excluding outpatient visits), based on electronic health records: Safely bonded parents panic less when an infant develops fever and may be more capable of

handling minor illnesses of their infants. A previous study [29] found a promising difference of 15% with music therapy compared to 30% with standard care.
(3) Infant *socio-emotional development* will be evaluated using the Ages and Stages Questionnaire Social-Emotional (ASQ:SE). This is a parent-completed questionnaire that targets social-emotional competence and problem behaviours, with 19 to 22 Likert-scaled items depending on child age [38, 39].
(4) Infant *social behaviour* will be assessed using the Alarm Distress Baby Scale (ADBB) [40]. It covers facial expression, eye contact, general level of activity, self-stimulating gestures, vocalizations, response to stimulation, relationship. It has shown high sensitivity, specificity and reliability [40].
(5) *Parental depression* will be assessed with the Edinburgh Postnatal Depression Scale (EPDS). The 10-item self-report instrument assesses caregiver's postpartum depressive symptoms, focusing specifically on more severe problems rather than those that are common to nearly all new mothers (such as loss of energy, feeling tired, changes in appetite and sexual drive). It has shown high sensitivity and specificity in two Norwegian studies [41, 42].
(6) Level of parental stress will be assessed with the Parental Stress Scale (PSS). This is a self-report 18-item questionnaire that assesses *stress levels associated with parenting* [43].
(7) In addition, a checklist of *medical and social factors* will be completed at baseline and updated 1-2 days prior to discharge, to track the presence of particular factors that might impact infant responsiveness (e.g., intraventricular hemorrhage, presence and nature of sedation, abnormal hearing screening at discharge, etc.), or parental engagement (e.g., socioeconomic status, receiving treatment for mental health or substance use problems, etc.). Within the MT group, physiological signs (heart rate, respiratory rate and oxygen saturation) and behavior state will be informally monitored during the course of MT as part of the provision of infant-directed approaches. This part will also assess use and costs of medical services. Existing electronic health records will be used wherever possible to improve reliability and completeness and to reduce costs and burdens of data collection.
Cost-effectiveness: We will collect the data that are necessary for analyzing cost-effectiveness both from a health services perspective (i.e. costs of all treatments incurred within the health sector) and from the broader societal perspective (i.e. including also indirect costs such as productivity losses). If clinical effectiveness is found, we will apply for separate funding to analyze incremental cost-effectiveness ratios and willingness to pay. Otherwise, these data will be used to describe the context in which the treatment took place.

2.2.4 Trial procedures, statistical considerations, and data management

Randomization, allocation concealment and blinding: After informed consent and baseline assessment, participants will be randomly assigned to NICU-MT or standard care using a computer-generated randomization list, with ratio 1:1, in blocks with sizes of 2 or 4 varying randomly, using email and an online system. The random allocation sequence will be generated and administered by people with no involvement in the clinical work to ensure allocation concealment. One day before discharge to home, participants will be randomized a second time to post-discharge MT or standard care in a 1:1 ratio, using the same procedures. This two-step randomization was chosen as the best way to create balance among those remaining in the study for the aftercare phase, and to avoid differential drop-out in the NICU phase due to expectations for the aftercare phase. In multiple pregnancies, only the first-born infant will be included and randomized, while remaining siblings will receive the same interventions for ethical and practical reasons. Blinding of interventions is not possible, but all outcomes that are not self-reports will be blinded, and success of blinding will be tested.
Power calculation and sample size: No previous RCT examined effects of MT on the PBQ; two small RCTs included in our systematic review [25] and a recent non-randomized study [29] examined mother-infant bonding, but provided insufficient data for meta-analysis. Studies using the PBQ with other interventions found effects ranging from around 0.25 ([44], posttest) through 0.5 ([45], mothers at 1/6 months), 1.4 ([46], 4 months), 2 ([45], fathers at 1/6 months), 9 ([44], 3 months), with SDs ranging from 4 to 12. Assuming a difference of 4 points on the PBQ (SD = 8) as a minimal clinically important difference for this study, power of 80% will be achieved for each main effect (each tested on a two-sided 2.5% significance level, i.e. 5% with Bonferroni correction for two tests) with a sample size of 155. Taking into account some clustering by country (ICC 0.01; 5 countries), this is increased to 203. To allow for 20% attrition, we will aim to include 250 infants (50 in each country) and their parents. This sample size will also ensure power for testing proportion differences of about 15% (e.g. binary analysis of problematic bonding; re-hospitalization). Special considerations are needed for multiple pregnancies (twins, triplets, etc.), which account for about 2% in Hordaland [4], but are more common in NICUs due to their elevated risk of preterm birth. Including all siblings would serve to increase the sample size, but may lower the resulting power due to cluster effects.

Therefore, we will only formally enroll and analyze the first-born sibling of each multiple pregnancy, although all will receive the intervention.

Data analyses: All randomized participants will be included in the main analysis (intention-to-treat). Descriptive statistics will characterize basic properties of the sample and provide information for power calculation for the multi-center study. Effects will be examined by testing the randomized groups for differences in the primary endpoint (t-test or Mann-Whitney; change in PBQ scores to 6 months CA), as well as by fitting linear mixed models. In the first modeling step we will assess the effect of the first randomization (Fig. 1) using the following model:

$PBQ_{it} = \beta_0 + \beta_1\, time_t + \beta_2\, gr_i + \beta_{12}\, time_t\, gr_i + U_i + \varepsilon_{it}$,

where PBQ_{it} denotes the PBQ score for participant i at measurement t, $time_t$ the time from baseline at measurement t and gr_i the randomization group of participant i. β_0, β_1, β_2 and β_{12} are the coefficients estimated in the model, U_i is an individual random term per participant and ε_{it} an independent, identically distributed error term. In the second step we refine the model to take into account both randomization steps, leading to the extended model:

$PBQ_{it} = \beta_0 + \beta_1\, time_t + \beta_2\, grR1_i + \beta_{12}\, time_t\, grR1_i + \beta_3\, grR2_i + \beta_{13}\, time_t\, grR2_i + U + \varepsilon_{it}$,

with the same notation as above and $grR1_i$ and $grR2i$ denoting the groups from the first and second randomization, respectively. In these models β_1 describes a group-independent time effect, β_2 and β_3 a time-independent group effect, and β_{12} and β_{13} the time-dependent change of the group effect. We will investigate both linear and simple contrasts (comparing each time point with the baseline) in time to assess the treatment effect. The same analyses will be done for the secondary outcomes; using an analogous generalized linear mixed model for binary outcomes. Per-protocol analyses will include those who received at least 6 MT sessions in the NICU and at least 5 MT sessions post-discharge, respectively; these are considered the minimum numbers for the intervention to be successful. Additionally, we will conduct exploratory statistical analyses for prespecified subgroups (sex; GA at birth [<28 wks, 28 to <32 wks, 32 to < 35wks]; hearing status at discharge [normal vs. abnormal]; bonding at baseline [PBQ impaired bonding score ≥ 11 vs. <11]) as well as qualitative/mixed-methods analyses of process data to maximize learning.

Data management plan: To promote transparency and applicability of trial results and to facilitate re-analysis by other researchers while also protecting anonymity, de-identified individual patient data will be made available through the Norwegian Centre for Research Data (NSD).

2.3 Applicability of the results of LongSTEP

The pragmatic nature of this trial and its high clinical applicability are reflected in several dimensions, as described in the pragmatic-explanatory continuum indicator summary (PRECIS) [47]. Eligibility criteria are designed to be as inclusive as possible to promote applicability of the evidence with respect to a broad population. Broad inclusion criteria ensure enrolment of participants with heterogeneous characteristics similar to those seen by clinicians in daily practice, and exclusion criteria are narrow and exist to ensure infant safety. Another pragmatic dimension of this trial is flexible MT delivered by a qualified music therapist. Instead of including infants in a narrow PMA range, we broaden the range of inclusion and instead tailor MT to infant PMA, while incorporating family and cultural preferences. Additionally, we do not restrict if or how co-interventions will be delivered. Both parallel groups will receive standard care. Analysis of primary outcome will include all individuals regardless of compliance in order to test if the intervention works under usual conditions, with all the noise inherent therein. As a limitation it should be noted that clinical benefits as well as resulting societal benefits, related to disability prevention and economic outcomes, are likely to occur over a longer-term perspective. To address this, we will also include the possibility in the consent forms to collect later follow-up data, but these will be beyond the present project. In summary, this pragmatic trial will determine the effects of MT under usual conditions, and the design as a multicenter trial will further ensure wide generalizability.

3. The project plan, project management, organization and cooperation

3.1 Project period, progress plan, main activities, milestones: see grant application form.

3.2 Project management, organization and cooperation

The project will draw upon Uni Research Health's multi-faceted experience in managing research projects. GAMUT – The Grieg Academy Music Therapy Research Centre, affiliated both to Uni Research Health (Uni Research's unit for health-related research) and the University of Bergen, provides an optimal context for both qualitative and quantitative research. GAMUT offers a multidisciplinary and international network

including an established MT research consortium of nine universities worldwide and the publication of two international, peer-reviewed MT journals. The Research Council of Norway evaluated GAMUT's scientific quality and relevance as "very good" (NFR, Evaluation Division for Science, 2011). Uni Research Health is specialized in conducting health-related research, with particular focus on RCTs including a pragmatic approach, as well as providing a strong base of methodological and statistical experts to support this project. The **core team** brings together researchers and clinicians with experience in multicenter RCTs as well as qualitative research, and experts in neonatal care across **three institutions in Bergen** (Uni Research [UniR], University of Bergen [UoB], Haukeland University Hospital [Haukeland]):

- **Christian Gold** (UniR, UoB), principal investigator (PI) for the first year: trained in music therapy, biostatistics, and trial management, with expertise in designing and conducting international multicenter RCTs of MT;
- **Łucja Bieleninik** (UniR), PI from the second year (presently on maternity leave): clinical psychologist and senior researcher, with experience in managing an international multicenter RCT of MT and expertise in the psychology of preterm infants and families;
- **Claire Ghetti** (UniR, UoB), associate professor and qualified NICU music therapist, with expertise in quantitative and qualitative research on MT in medical areas;
- **Hanne Cecilie Braarud** (UniR), senior researcher and clinical psychologist with expertise in quantitative research and observational analyses in infant mental health and infant development;
- **Bente Vederhus** (Haukeland), nurse leader, neonatal intensive care nurse and qualified NIDCAP observer, with experience in long-term follow-up studies of extremely preterm infants;
- along with **Brynjulf Stige** (UniR, UoB), head of GAMUT, professor in music therapy with extensive experience in service development and implementation; **Jörg Assmus** (UniR, Haukeland), project statistician, with experience in analyzing international RCTs of MT; **Jo Wake** (UniR)**,** information scientist with experience in database development for RCTs; and a music therapist clinician-researcher position (PhD or postdoc).

This interdisciplinary team, experienced in conducting high-quality RCTs in MT research, will be responsible for the overall conduct and integrity of the study. In addition to the three institutions above, the network of ***national and international partners*** includes a range of clinical, research, and user organizations. ***Clinical sites*** were chosen with respect to similarity of health care systems, society, and culture (i.e. representing a social support society where parents are consistently present in the NICU) and with regard to relevant scientific and clinical expertise; all have confirmed their intent to collaborate:

- Haukeland University Hospital, Bergen, Norway (Thomas Halvorsen, research coordinator, Department of Pediatrics; Hallvard Reigstad, neonatologist; Bente Vederhus, intensive care nurse)
- Akershus University Hospital, Oslo, Norway (Anne Lee Solvåg, substitute head of department for research and development and medical director; Tora Gaden, music therapist)
- Oslo University Hospital, Oslo, Norway (Arild Rønnestad, head of medical branch, Rikshospitalet; Julie Mangersnes, music therapist)
- Central Hospital Karlstad, Sweden (Maria Lindström Bagge, department director; Alexandra Ullsten, music therapist)
- Charité Universitätsmedizin Berlin, Germany (Christoph Bührer, director of Neonatology; Stephanie Scileppi, music therapist)
- Meir Medical Center, Tel Aviv University, Israel (Shmuel Arnon, deputy head of Neonatology; Dana Yakobson, music therapist; Cochavit Elefant, music therapist and director of the Graduate School of Creative Arts Therapies at Haifa University)
- Clinica de la Mujer, Bogotá, Colombia (Monica A. Cuevas Eslava, medical director; Mark Ettenberger, coordinator of music therapy)

Recruitment strategy: The sites listed above vary between 13 and 60 beds and between 250 and 500 admissions per year. Following a pilot cohort at one site, an 18-month period for recruitment will be sufficient to achieve the targeted sample size. Each site will aim to recruit about 50 infants and their parents; therefore, slow recruitment at some sites will be tolerable. Recruitment rates will be monitored carefully. Local and national partners responsible for primary care provision, such as Bergen Municipality in the Bergen area, will serve as contact points after discharge and will be actively involved wherever possible. ***Other partners:*** King's College London, UK (Renee Romeo, senior lecturer, King's Health Economics) will be responsible for service use and costs with a view towards evaluating cost-effectiveness.

CVs of the most central researchers in the team are enclosed, demonstrating the range of expertise relevant to the project. Letters of intent from partners have been collected but are not enclosed.
As a ***user representative***, Trude Os, mother of two preterm infants and connected to Prematurforeningen, will help to ensure the project's user relevance. An ***international scientific advisory panel*** of clinicians and researchers with relevant clinical expertise will include Friederike Haslbeck (University Hospitals Zurich and Bern, Switzerland); Helen Shoemark (Temple University, USA); Deanna Hanson-Abromeit (University of Kansas, USA); Małgorzata Lipowska (University of Gdansk, Poland); Joanne Loewy (Mount Sinai Beth Israel and Icahn School of Medicine at Mount Sinai, USA); and Renate Nussberger (Kantonsspital Baden, Switzerland). An ***independent Data and Safety Monitoring Committee*** consisting of clinicians and methodologists with relevant expertise but no direct involvement in the trial will be established to ensure research integrity and safety of participants in the trial, and will recommend appropriate action if ethical issues arise.
3.3 Budget: see grant application form. Contributions to partner sites are primarily through per-patient costs, adjusted to the price level of each country; this provides a fair and balanced allocation of resources and helps to boost recruitment.

4. Key perspectives and compliance with strategic documents

4.1 Compliance with strategic documents*:* Research relevant to music and health is a focus area of Uni Research Health, and high-quality RCTs that can inform health services are the mainstay of its research agenda. This project improves caregiver sensitivity to premature infant signals and needs, and supports development of healthy infant/parent relations during the postnatal period consistent with national guidelines [31].
4.2 Relevance and benefit to society*:* The project's social impact includes filling gaps in knowledge regarding the long-term impact of MT with preterm infants/caregivers, including impact on the highly user-relevant outcomes of bonding, infant development, and parental psychological health. The high applicability of this trial will facilitate ongoing development of evidence-based care for preterm infants. The project makes a unique contribution to continuity of care, by bridging intervention from NICU hospitalization to municipal and home settings post-discharge. Findings will impact families and children in a high-risk group, as well as music therapists, psychologists, medical staff, and municipal health care workers. Dissemination of the findings will apply to local, national, and international levels.
4.3 Environmental impact: not applicable.
4.4 Ethical perspectives: Ethical approval will be secured before start of recruitment from the Regional Committee for Medical Research Ethics in Western Norway (REK Vest), as well as data security clearance from NSD, and corresponding bodies in the other participating countries. The study will be registered in the ISRCTN database before the first patient's first visit. Informed consent will be obtained postnatally following routine practices in the NICU. Trial staff will inform parents/caregivers about consent for participation. The parent/caregiver will receive written and oral explanation of the proposed research project including aims, duration of involvement, expected benefits to participants and others, nature of the interventions, procedures involved in participation, and any potential risks. It will be emphasized that enrolment in the study is voluntary, that parent/caregivers can withdraw at any time from all or part of the study, and that any decision they take in this respect will have no bearing on the care the infant and family receive. Participants will be informed that the outcomes of the study will be published, but that no details will be divulged from which the participant could be identified. The primary caregivers will also be informed who to contact in an emergency and how to discontinue participation if desired. The consent form will be available in Norwegian and English, and other participating countries will create translations as needed. All significant harms as well as unintended effects for each group will be collected and described. Special forms to report trial-related adverse events will be developed, and all professionals involved in the trial will be informed about how to identify and report adverse events. Any serious adverse effects that occur will be reported, and if related to MT, will be reported to the DMC.
4.5 Gender issues (Recruitment of women, gender balance and gender perspectives): Both boys and girls born prematurely will be included. Primary caregivers of both genders will be included in this trial, although we anticipate that predominantly mothers will be participating in MT sessions. The gender distribution of researchers in the core team and among collaborators is balanced. The role of PI will be handed to a female researcher after the first year (see above).

5. Dissemination and communication of results

5.1 Dissemination plan – see grant application form.

5.2 Communication with users: The most important users are families with preterm infants. To ensure the integrity and quality of the research project, we will include both mothers and fathers of preterm infants in project planning, implementation and evaluation. We have engaged a user representative from Prematurforeningen for development of the proposal to assure relevance to users within the Norwegian context, and will encourage our partner countries to do the same to identify user representative who can inform all stages of the project. User representatives will be invited to meetings to provide feedback on design aspects, results and experiences; to present their view at public presentations; and to help disseminating study findings. Additional users include hospitals, researchers, and policy makers. We elicited feedback from our participating Norwegian hospitals and international advisory network during the formation of this proposal.

6. Additional information requested in the call

This project fosters **interdisciplinary collaboration** between **specialist and primary/municipal health care services** (psychologists, nurses, physicians, music therapists); uniting a high-powered MT research center with researchers and clinicians at local, national and international levels. The **user involvement** for this study (see separate text) provides a structured avenue for eliciting user feedback and ensuring relevance and efficiency in adjustments of the research protocol, ongoing evaluation of the research, and dissemination of findings. A user representative from Prematurforeningen of Norway has already been involved, and similar involvement of users in other countries will be encouraged. Qualitative feedback from study participants regarding their experiences of completing study procedures and measures will also help to provide insight on user perspectives. This project **builds off of evidence-based practice in neonatal MT**, which supports the use of infant-specific MT tailored to developmental level and provided in conjunction with primary caregivers. The project **advances previous knowledge** by suggesting a pathway by which MT can promote infant development while improving parental psychological outcomes. Coaching parents in the use of NICU-MT (as described in this protocol) can help improve infant regulation and decrease parental stress during parent/infant interactions, which may promote bonding and contribute to better infant developmental and parental psychological outcomes. NICU-MT used in this study consists of a relatively high-intensity infant/parent interaction protocol, which is feasible within a Norwegian context, and in similar contexts worldwide where parents are highly present throughout NICU hospitalization. Results of a recent systematic review of RCTs evaluating MT for preterm infants identified lack of longitudinal studies as a major gap in knowledge [25]. This project directly **addresses that gap** by assessing the longitudinal impact of MT on preterm infants and caregivers across a 12-month period. The outcomes of this project will be directly **applicable to clinical practice** in Norway, a country that is just beginning to develop practice in NICU MT and to train music therapists to work appropriately and effectively with preterm infants and families.

References (Publications by team members are marked with*)

1. Blencowe, H., et al., *Born too soon: the global epidemiology of 15 million preterm births.* Reprod Health, 2013. **10**(Suppl 1:S2).
2. Blencowe, H., et al., *National, regional, and worldwide estimates of preterm birth rates in the year 2010 with time trends since 1990 for selected countries: a systematic analysis and implications.* Lancet, 2012. **379**(9832): p. 2162-2172.
3. Liu, L., et al., *Global, regional, and national causes of child mortality: an updated systematic analysis for 2010 with time trends since 2000.* Lancet, 2012. **379**(9832): p. 2151-2161.
4. Duerr, A., et al., *Årstabeller for Medisinsk fødselsregister 2012. Fødsler in Norge.* 2014: Utgitt av Folkehelseinstituttet.
5. Underwood, M.A., B. Danielsen, and W.M. Gilbert, *Cost, causes and rates of rehospitalization of preterm infants.* J Perinatol, 2007. **27**(10): p. 614-619.
6. Singer, L.T., et al., *Maternal psychological distress and parenting stress after the birth of a very low-birth-weight infant.* JAMA, 1999. **281**(9): p. 799–805.
7. Jotzo, M. and C.F. Poets, *Helping parents cope with the trauma of premature birth: an evaluation of a trauma-preventive psychological intervention.* Pediatrics, 2005. **115**(4): p. 915-919.
8. * Bieleninik, Ł., M. Bidzan, and D. Salwach, *The premature birth trauma and the parents' quality of life in the light of the S. Allen and A. Michalos' models*, in *Wellness and success* O. G, Editor. 2009, NeuroCentrum: Lublin. p. 39–52.
9. * Bieleninik, Ł., *Dzieci urodzone przedwcześnie w percepcji matek [Mothers' perception of prematurely born children in the period of the early childhood]*. 2012, Gansk: Harmionia Universalis.
10. * Bidzan, M., et al., *Bond with a child in the prenatal period in case of prematurely born children*, in *Wellness and success* K. Turowski, Editor. 2009, NeuroCentrum: Lublin. p. 35–54.
11. Bowlby, J., *Attachment and loss: Vol 1*. 1997, London: Pimlico.

12. Ainsworth, M.D.S., et al., *Patterns of attachment: A psychological study of the strange situation*. 1978, Hillsdale NJ: Erlbaum.
13. Korja, R., et al., *Maternal depression is associated with mother–infant interaction in preterm infants.* Acta Paediatr, 2008. **97**(6): p. 724–730.
14. Muller-Nix, C., et al., *Prematurity, maternal stress and mother-child interactions.* Early Hum Dev, 2004. **79**(2): p. 145-158.
15. Korja, R., R. Latva, and L. Lehtonen, *The effects of preterm birth on mother-infant interaction and attachment during the infant's first two years.* Acta Obstet Gynecol Scand, 2012. **91**(2): p. 164-173.
16. * Ghetti, C.M., *Pediatric intensive care*, in *Guidelines for music therapy practice in pediatric care* J. Bradt, Editor. 2013, Barcelona Publishers: Gilsum, NH. p. 152-204.
17. Haslbeck, F.B., *Music therapy for premature infants and their parents: an integrative review.* Nord J Music Ther, 2012. **21**(3): p. 203-226.
18. Standley, J.M. and D. Walworth, *Music therapy with premature infants: research and developmental interventions (2nd ed.).* 2010, Silver Spring, MD: American Music Therapy Association.
19. Whipple, J., *The effect of parent training in music and multimodal stimulation on parent–neonate interactions in the neonatal intensive care unit.* J Music Ther, 2000. **37**(4): p. 250–268.
20. Hartling, L., et al., *Music for medical indications in the neonatal period: a systematic review of randomised controlled trials.* Arch Dis Child Fetal Neonatal Ed, 2009. **94**(5): p. 349-354.
21. Loewy, J., et al., *The effects of music therapy on vital signs, feeding, and sleep in premature infants.* Pediatrics, 2013. **131**: p. 902-918.
22. Standley, J.M., *Music Therapy for the Neonate.* Newborn Infant Nurs Rev, 2001. **1**(4): p. 211-216.
23. Standley, J.M., *A meta-analysis of the efficacy of music therapy for premature infants.* J Pediatr Nurs, 2002. **17**(2): p. 107-113.
24. Standley, J.M., *Music therapy research in the NICU: an updated meta-analysis.* Neonatal Netw, 2012. **31**(5): p. 311-316.
25. * Bieleninik, Ł., C. Ghetti, and C. Gold, *Music therapy for preterm infants and their parents: A meta-analysis.* Pediatrics, 2016. **138**(3).
26. Haslbeck, F.B., *The interactive potential of creative music therapy with premature infants and their parents: a qualitative analysis.* Nord J Music Ther, 2014. **23**(1): p. 36-70.
27. * Stormark, K.M. and H.C. Braarud, *Infants' sensitivity to social contingency: a "double video" study of face-to-face communication between 2- and 4-month-olds and their mothers.* Infant Behav Dev, 2004. **27**: p. 195-203.
28. Malloch, S. and C. Trevarthen, eds. *Communicative musicality: exploring the basis of human companionship*. 2009, Oxford University Press: Oxford.
29. * Ettenberger, M., et al., *Family-centered music therapy with preterm infants and their parents in the neonatal intensive care unit (NICU) in Colombia -- a mixed methods study.* Nord J Music Ther, 2017. **26**(3): p. 207-234.
30. American Academy of Pediatrics, *Age terminology during the perinatal period.* Pediatrics, 2004. **114**(5): p. 1362-1364.
31. Markestad, T. and B. Halvorsen, *Faglige retningslinjer for oppfølging av for tidlig fødte barn*, 2007, Sosial- og helsedirektoratet: Oslo.
32. Brockington, I.F., et al., *A Screening Questionnaire for mother-infant bonding disorders.* Arch Womens Ment Health, 2001. **3**(4): p. 133-140.
33. Hoffenkamp, H.N., et al., *The impact of premature childbirth on parental bonding.* Evol Psychol, 2012. **10**(3): p. 542-561.
34. Perrelli, J.G., et al., *Mother-child bonding assessment tool.* Rev Paul Pediatr, 2014. **32**(3): p. 257−265.
35. Wittkowski, A., J. Williams, and A. Wieck, *An examination of the psychometric properties and factor structure of the Post-partum Bonding Questionnaire in a clinical inpatient sample.* Br J Clin Psychol, 2010. **49**: p. 163–172.
36. Hoivik, M.S., et al., *The Mother and Baby Interaction Scale: a valid broadband instrument for efficient screening of postpartum interaction? A preliminary validation in a Norwegian community sample.* Scand J Caring Sci, 2013. **27**(3): p. 733-739.
37. Bayley, N., *Bayley Scales of Infant and Toddler Development, 3rd edition*, ed. Pearson. 2006, San Antonio: Pearson.
38. Yovanoff, P. and J. Squires, *Determining cutoff scores on a developmental screening measure: Use of receiver operating characteristics and item response theory.* J Early Interv, 2006. **29**(1): p. 48-62.
39. Squires, J., et al., *Identification of social-emotional problems in young children using a parent-completed screening measure.* Early Child Res Q, 2001. **16**(4): p. 405-419.
40. Guedeney, A. and J. Fermanian, *A validity and reliability study of assessment and screening for sustained withdrawal reaction in infancy: The alarm distress baby scale.* Infant Ment Health J, 2001. **22**(5): p. 559-575.
41. Eberhard-Gran, M., et al., *The Edinburgh Postnatal Depression Scale: validation in a Norwegian community sample.* Nord J Psychiatry, 2001. **55**(2): p. 113-117.
42. Berle, J.Ø., et al., *Screening for postnatal depression: Validation of the Norwegian version of the Edinburgh Postnatal Depression Scale, and assessment of risk factors for postnatal depression.* J Affect Disord, 2003. **76**(1-3): p. 151-156.
43. Berry, J.O. and W.H. Jones, *The parental stress scale: initial psychometric evidence.* J Soc Pers Relat, 1995. **12**(3): p. 463-472.
44. Tsivos, Z.-L., et al., *A pilot randomised controlled trial to evaluate the feasibility and acceptability of the Baby Triple P Positive Parenting Programme in mothers with postnatal depression.* Clin Child Psychol Psychiatry, 2015. **20**(4): p. 532-554.
45. Hoffenkamp, N.H., et al., *Effectiveness of hospital-based video interaction guidance on parental interactive behavior, bonding, and stress after preterm birth: a randomized controlled trial.* J Consult Clin Psychol, 2015. **83**(2): p. 416-429.
46. O'Mahen, H.A., et al., *Netmums: a phase II randomized controlled trial of a guided Internet behavioural activation treatment for postpartum depression.* Psychol Med, 2014. **44**(8): p. 1675-1689.
47. Thorpe, K.E., et al., *A pragmatic-explanatory continuum indicator summary (PRECIS): a tool to help trial designers.* J Clin Epidemiol, 2009. **62**(5): p. 464-475.

Summary of amendments to the original study protocol

Domain	Original wording	Revised to	Rationale
Eligibility criteria: parents	"…primary caregivers should live within reasonable commuting distance from the treating NICU in order to be eligible for participation." (Did not specify parental capacity to engage in treatment or assessments).	Added specification: "Primary caregivers should live within reasonable commuting distance from the treating NICU and should have sufficient understanding of the respective national language(s) to answer the questionnaires and participate in MT. Parents who are unable to complete the intervention and/or questionnaires, for example, due to a mental illness, cognitive impairment and so on, will be excluded."<br><br>[Date of change 1 September 2108]	To further specify inclusion and exclusion criteria related to parental capacity to engage in treatment and/or assessments.

Summary of amendments to the published study protocol

Domain	Original wording	Revised to	Rationale
Secondary infant outcome: child development	"the most important areas for the present study will be language and cognition"	Primary outcome was language development (BSID-III language composite score), with remaining BSID-III composite and subscale scores as secondary outcomes.	Between language and cognitive domains, we found language to be most relevant for this study; therefore, we pre-specified the language composite score as a primary outcome
Time point	at 24 months child corrected age	extended to 32 months corrected age	Due to COVID-19 related challenges to make assessments more feasible
