# Supplementary material for: Music Therapy in Infancy and Neurodevelopmental Outcomes in Preterm Children: A Secondary Analysis of the LongSTEP Randomized Clinical Trial
Source: JAMA Netw Open. 2024 May 16;7(5):e2410721. doi: 10.1001/jamanetworkopen.2024.10721 (PMC11099691; doi:10.1001/jamanetworkopen.2024.10721)
Supplement: Supplement 2. — eAppendix. Standardization Procedures of Neurodevelopmental Assessment With the Bayley Scales of Infant and Toddler Development, 3rd Edition (BISD-III) eTable 1. Infant Baseline Characteristics of Those Followed-Up Versus Lost to Follow-Up eTable 2. Completers by Country and Intervention Group eTable 3. Clinical and Demographic Predictors of the Bayley Scales of Infant and Toddler Development, 3rd Edition (BISD-III) Language Composite Score eFigure 1. Estimates of Clinical and Demographic Predictors of the Bayley Scales of Infant and Toddler Development, 3rd Edition (BISD-III) Language Composite Score eTable 4. Intervention Effects by Selected Clinical/Demographic Predictors of the Bayley Scales of Infant and Toddler Development, 3rd Edition (BISD-III) Language Composite Score eTable 5. Sensitivity Analysis: Estimates of Intervention Effects on the Bayley Scales of Infant and Toddler Development, 3rd Edition (BISD-III) Language Composite Score Using Multiple Imputation eFigure 2. Estimates of Intervention Effects on the Bayley Scales of Infant and Toddler Development, 3rd Edition (BISD-III) Language Composite Score by Selected Clinical/Demographic Predictors eFigure 3. Effects of Music Interventions on the Bayley Scales of Infant and Toddler Development, 3rd Edition (BISD-III) at 24 Months Across Studies eReferences [file jamanetwopen-e2410721-s002.pdf]

Supplemental Online Content

Bieleninik Ł, Kvestad I, Gold C, et al. Music therapy in infancy and neurodevelopmental outcomes in preterm children: a secondary analysis of the LongSTEP randomized clinical trial. *JAMA Netw Open*. 2024;7(5):e2410721. doi:10.1001/jamanetworkopen.2024.10721

eAppendix. Standardization Procedures of Neurodevelopmental Assessment With the Bayley Scales of Infant and Toddler Development, 3rd Edition (BISD-III)

eTable 1. Infant Baseline Characteristics of Those Followed-Up Versus Lost to Follow-Up

eTable 2. Completers by Country and Intervention Group

eTable 3. Clinical and Demographic Predictors of the Bayley Scales of Infant and Toddler Development, 3rd Edition (BISD-III) Language Composite Score

eFigure 1. Estimates of Clinical and Demographic Predictors of the Bayley Scales of Infant and Toddler Development, 3rd Edition (BISD-III) Language Composite Score

eTable 4. Intervention Effects by Selected Clinical/Demographic Predictors of the Bayley Scales of Infant and Toddler Development, 3rd Edition (BISD-III) Language Composite Score

eTable 5. Sensitivity Analysis: Estimates of Intervention Effects on the Bayley Scales of Infant and Toddler Development, 3rd Edition (BISD-III) Language Composite Score Using Multiple Imputation

eFigure 2. Estimates of Intervention Effects on the Bayley Scales of Infant and Toddler Development, 3rd Edition (BISD-III) Language Composite Score by Selected Clinical/Demographic Predictors

eFigure 3. Effects of Music Interventions on the Bayley Scales of Infant and Toddler Development, 3rd Edition (BISD-III) at 24 Months Across Studies

eReferences

This supplemental material has been provided by the authors to give readers additional information about their work.

eAppendix. Standardization procedures of neurodevelopmental assessment with the Bayley Scales of Infant and Toddler Development, 3rd edition (BISD-III)

The standardization procedures prior to assessments were adjusted to the assessors' level of experience. Those with no prior experience with BSID-III assessments (Poland) took part in five standardization exercises along with a video recording of an assessment evaluated by a project senior psychologist. In the standardization procedures, the assessors administered the test with a child at approximately 24 months while the other assessor observed and double scored. The assessors were ready to start with assessments for the LongSTEP study when an inter-rater agreement >0.9 was reached in the standardization exercises and the video was approved by the project senior psychologists. Assessors with experience in BSID-III assessments (Argentina, Israel, Colombia and Norway) did not take part in these standardization procedures.

© 2024 Bieleninik Ł et al. *JAMA Network Open*

eTable 1. Infant baseline characteristics of those followed-up versus lost to follow-up

	Followed up at 24 months		Not followed up		Test for difference
Baseline characteristic	N	No. (%)	N	No. (%)	p-value
**Sex (female)**	111	59 (53)	95	44 (46)	0.4017[2]
**Singleton pregnancy**	111	83 (75)	95	62 (65)	0.181[2]
**Cesarean delivery route**	111	87 (78)	95	81 (85)	0.2758[2]
**Birth weight (grams), mean (sd)**	111	1382 (414) [480, 2335]	94	1423 (456) [590, 2440]	0.5064[3]
**GA at birth (weeks), mean (sd)**	111	30.41 (2.59) [22.86, 34.29]	95	30.56 (2.73) [22.86, 34.71]	0.6875[3]
**GA <28 weeks**		20 (18)		16 (17)	0.3852[4]
**GA 28-32 weeks**		51 (46)		36 (38)	
**GA 32-35 weeks**		40 (36)		43 (45)	
**PMA enrollment (weeks), mean (sd)**	111	33.05 (1.86) [27.43, 37.57]	95	33.1 (2.08) [28.71, 44.00]	0.8711[3]
**Apgar score at 5min, mean (sd)**	109	8.7 (1.42) [1, 10]	92	8.5 (1.25) [5, 10]	0.5064[3]
**Weight at enrollment (grams), mean (sd)**	109	1614 (380) [705, 2730]	94	1626 (460) [759, 3680]	0.8466[3]
**Estimated severity of IVH[1]**	111		95		0.2825[4]
Cranial Ultrasound not indicated		43 (39)		44 (46)	
None		53 (48)		44 (46)	
Grade 1-2		15 (13)		4 (5)	
Grade 3-4		0 (0)		3 (3)	

Abbreviations: SD, standard deviation; GA, gestational age; PMA, post-menstrual age; IVH, intraventricular hemorrhage [1] IVH was diagnosed by cranial ultrasound and graded according to Papile et al.[1] [2] From two-sided Z-test; [3] From two-sided t-test; [4] From chi square test

© 2024 Bieleninik Ł et al. *JAMA Network Open*

eTable 2. Completers by country and intervention group

	SCSC	MTSC	SCMT	MTMT	Total
**ARG**	0/2 (0%)	1/5 (20%)	1/5 (20%)	0/2 (0%)	14
**COL**	2/18 (11%)	7/15 (47%)	6/16 (38%)	11/18 (61%)	67
**ISR**	10/13 (77%)	10/14 (71%)	13/14 (93%)	9/13 (69%)	54
**NOR**	6/7 (86%)	6/8 (75%)	4/9 (44%)	6/9 (67%)	33
**POL**	6/10 (60%)	5/9 (56%)	2/9 (22%)	5/10 (50%)	38
**Total**	50	51	53	52	206

Abbreviations: SCSC, Standard care Standard care; MTSC, Music therapy Standard care; SCMT, Standard care Music therapy; MTMT, Music therapy Music therapy; ARG, Argentina; COL, Colombia; ISR, Israel; NOR, Norway; POL, Poland
Note. Showing completers (valid BSID-III Language Composite Score), totals, and percentage of completers by country and intervention group. No significant association of completers between country and intervention group was found (Fisher's Exact Test for Count Data, p = 0.468).

© 2024 Bieleninik Ł et al. *JAMA Network Open*

eTable 3. Clinical and demographic predictors of the Bayley Scales of Infant and Toddler Development, 3rd edition (BISD-III) Language Composite score

	Language Composite		
Predictors	*Estimates*	*CI*	*p*
(Intercept)	80.62 **	26.67 – 134.58	0.004
GA at birth	0.71	-0.99 – 2.41	0.408
Male sex	-9.30 *	-17.38 – -1.22	0.025
Skin-to-skin care until discharge ≥ 4 days/week	5.19	-9.63 – 20.02	0.488
University degree mother	0.19	-10.65 – 11.04	0.972
University degree father	0.03	-8.95 – 9.01	0.994
Caeserean delivery	-1.20	-10.72 – 8.31	0.802
Multiple birth	0.38	-8.80 – 9.55	0.935
Oxygen supplementation before discharge	-18.48 *	-33.93 – -3.02	0.020
Abnormal head ultrasound before discharge	6.41	-6.02 – 18.85	0.308
**Random Effects**			
σ^2	362.55		
$\tau_{00\ site}$	6.19		
N_{site}	8		
Observations	98		
Marginal R^2 / Conditional R^2	0.167 / NA		

* *p<0.05* ** *p<0.01* *** *p<0.001*

Note. Based on a linear mixed-effects model with site as random effects. Effects of oxygen supplementation were also explored by country (Colombia versus others). Oxygen supplementation was very common at the Colombian site because its high altitude. It did not predict scores there, thus masking an even larger effect when Colombia was removed (not shown).

© 2024 Bieleninik Ł et al. *JAMA Network Open*

eFigure 1. Estimates of clinical and demographic predictors of the Bayley Scales of Infant and Toddler Development, 3rd edition (BISD-III) Language Composite score

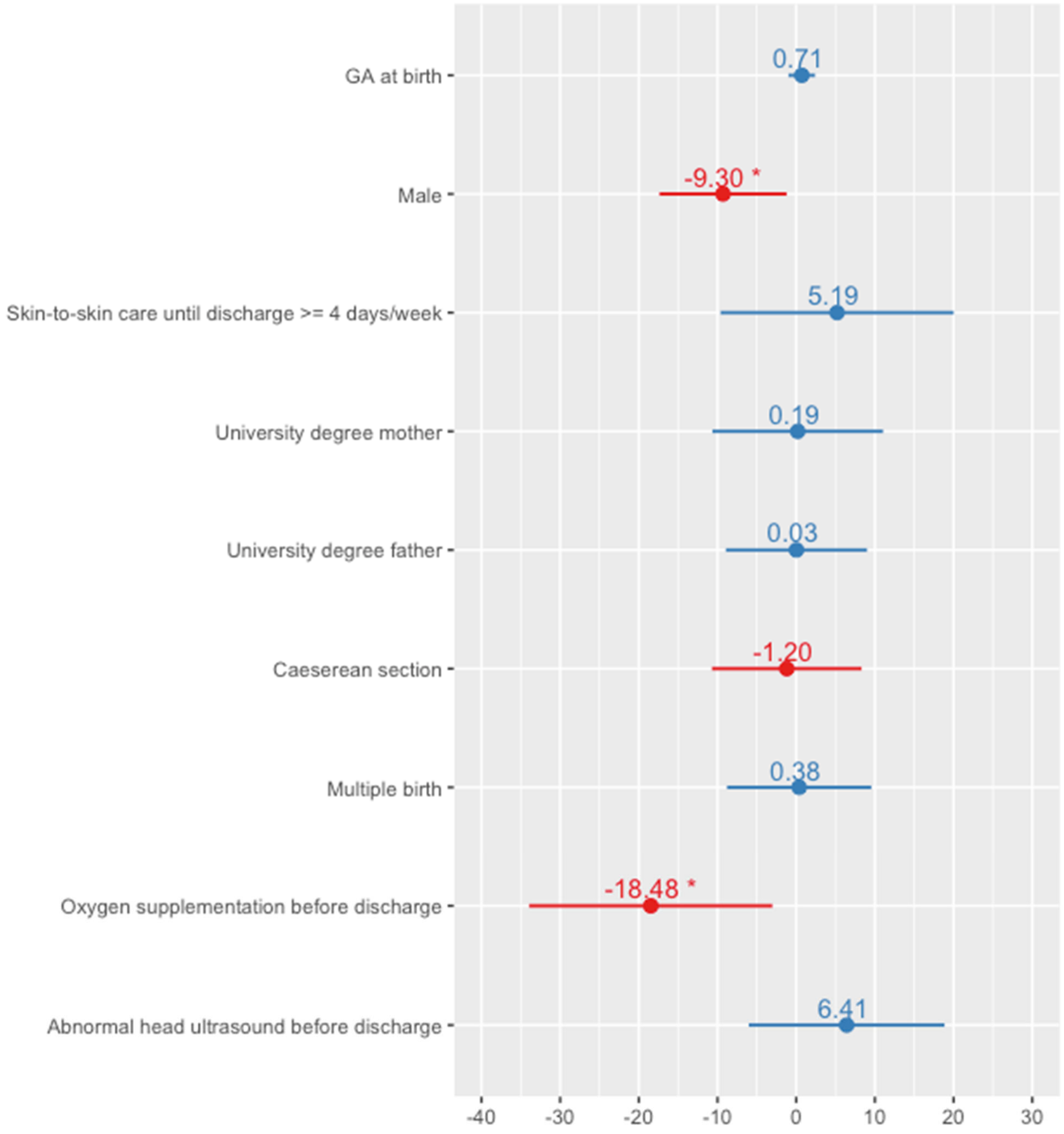

Note. Graphical display of the estimates in the model presented in eTable 3.

© 2024 Bieleninik Ł et al. *JAMA Network Open*

eTable 4. Intervention effects by selected clinical/demographic predictors of the Bayley Scales of Infant and Toddler Development, 3rd edition (BISD-III) Language Composite score

	Language Composite		
Predictors	*Estimates*	*CI*	*p*
(Intercept)	100.31 ***	89.56 – 111.05	<0.001
MTSC	1.77	-12.38 – 15.92	0.804
SCMT	3.53	-11.38 – 18.45	0.639
MTMT	2.28	-13.04 – 17.59	0.769
Male sex	-6.64	-21.46 – 8.17	0.375
Oxygen supplementation before discharge	3.40	-23.47 – 30.26	0.802
MTSC X Male sex	-5.01	-25.33 – 15.31	0.625
SCMT X Male sex	3.52	-17.08 – 24.12	0.735
MTMT X Male sex	-3.43	-23.29 – 16.43	0.732
MTSC X Oxygen suppl.	-18.90	-50.12 – 12.32	0.232
SCMT X Oxygen suppl.	-19.25	-50.94 – 12.43	0.231
MTMT X Oxygen suppl.	-23.01	-53.31 – 7.28	0.135
**Random Effects**			
σ^2	333.58		
$\tau_{00\ site}$	0.78		
ICC	0.00		
N_{site}	8		
Observations	110		
Marginal R^2 / Conditional R^2	0.182 / 0.184		

* *p<0.05* ** *p<0.01* *** *p<0.001*

Abbreviations: MTSC, Music therapy Standard care; SCMT, Standard care Music therapy; MTMT, Music therapy Music therapy, ICC, Intraclass correlation; N, Number

Note. Based on a linear mixed-effects model, selecting significant predictors from eTable 3 for interaction with intervention group.

© 2024 Bieleninik Ł et al. *JAMA Network Open*

eTable 5. Sensitivity analysis: Estimates of intervention effects on the Bayley Scales of Infant and Toddler Development, 3rd edition (BISD-III) Language Composite score using multiple imputation

	Language Composite		
Predictors	*Estimates*	*SE*	*p*
(Intercept)	91.52 ***	5.26	<0.001
MTSC	-0.54	4.15	0.897
SCMT	2.51	4.97	0.619
MTMT	-3.61	4.30	0.406

* *p<0.05* ** *p<0.01* *** *p<0.001*

Abbreviations: MTSC, Music therapy Standard care; SCMT, Standard care Music therapy; MTMT, Music therapy Music therapy
Note. Pooled summary of linear mixed-effects models from 5 complete datasets generated with multiple imputation (R package mice, using predictive mean matching for continuous variables and logistic regression for binary variables; including selected clinical/demographic variables, site, intervention group, and all outcomes in the imputation).

© 2024 Bieleninik Ł et al. *JAMA Network Open*

eFigure 2. Estimates of intervention effects on the Bayley Scales of Infant and Toddler Development, 3rd edition (BISD-III) Language Composite score by selected clinical/demographic predictors

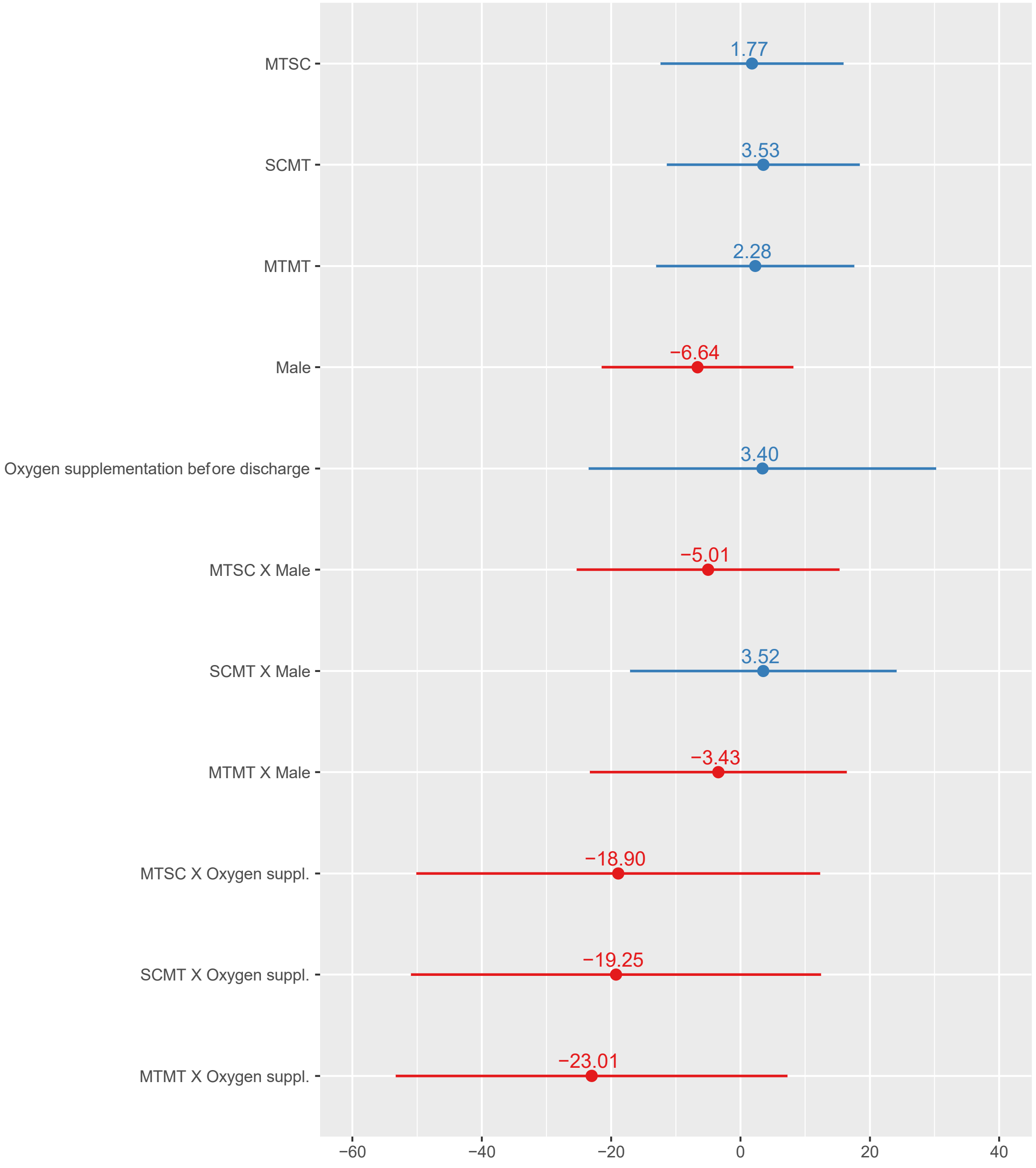

Note. Graphical display of the estimates in the model presented in eTable 4.

© 2024 Bieleninik Ł et al. *JAMA Network Open*

eFigure 3. Effects of music interventions on the Bayley Scales of Infant and Toddler Development, 3rd edition (BISD-III) at 24 months across studies

a) Language Composite

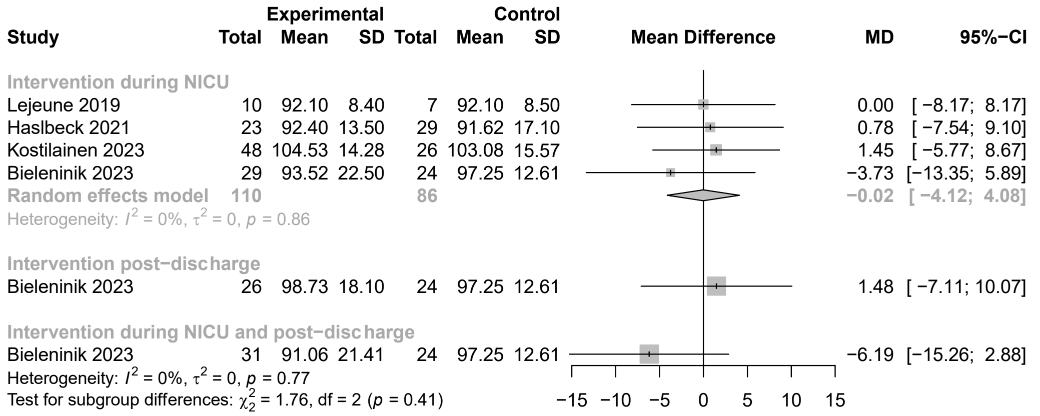

b) Motor composite

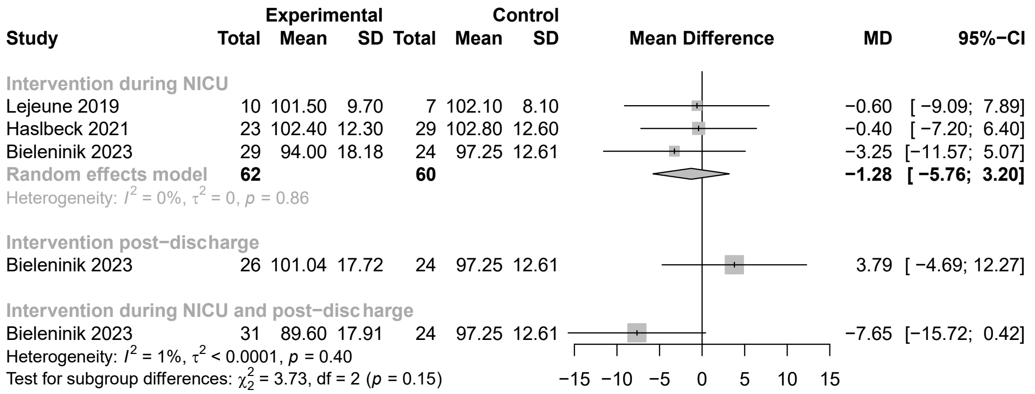

c) Cognitive Composite

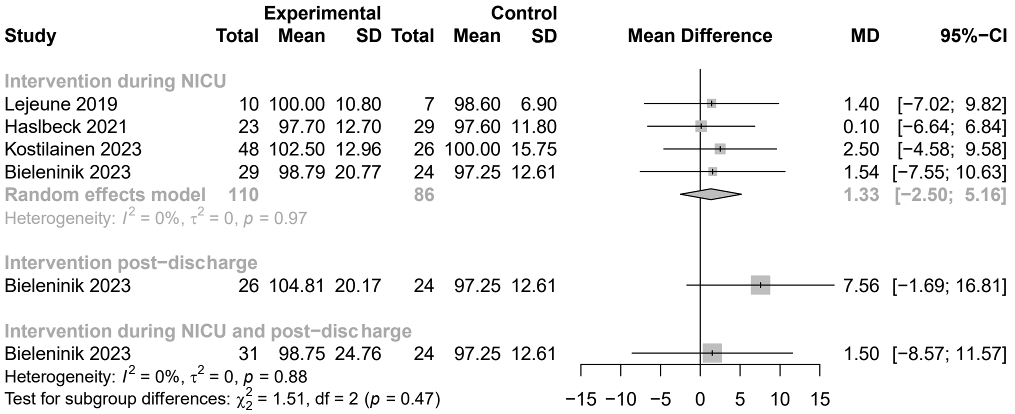

Note: We present random-effects meta-analyses because of high clinical heterogeneity between interventions. Lejeune et al. [2] used music listening to recorded music without a music therapist (tracks were created by a nurse); Haslbeck et al. [3] used creative MT where the music therapist led the intervention; Bieleninik et al (present study) used parent-led, infant-directed singing predominantly led by the parent. End

© 2024 Bieleninik Ł et al. *JAMA Network Open*

of intervention post-discharge refers to 6 months in Bieleninik et al. Effects of intervention during NICU and post–discharge refers to music therapist provided 3 times weekly in NICU and 7 sessions post-NICU across 6 months in Bieleninik et al.

© 2024 Bieleninik Ł et al. *JAMA Network Open*

eReferences

1. Papile LA, Burstein J, Burstein R, Koffler H. Incidence and evolution of subependymal and intraventricular hemorrhage: a study of infants with birth weights less than 1,500 gm. *The Journal of Pediatrics*. 1978;92(4):529–534. doi:10.1016/s0022-3476(78)80282-0

2. Lejeune F, Lordier L, Pittet MP, et al. Effects of an Early Postnatal Music Intervention on Cognitive and Emotional Development in Preterm Children at 12 and 24 Months: Preliminary Findings. *Frontiers in Psychology*. 2019;10(494)doi:10.3389/fpsyg.2019.00494

3. Haslbeck FB, Bucher HU, Bassler D, Hagmann C, Natalucci G. Creative Music Therapy and Neurodevelopmental Outcomes in Pre-term Infants at 2 Years: A Randomized Controlled Pilot Trial. *Frontiers in Pediatrics*. 2021;9:660393. doi:10.3389/fped.2021.660393
