# Supplementary material for: Music Therapy in Infancy and Neurodevelopmental Outcomes in Preterm Children: A Secondary Analysis of the LongSTEP Randomized Clinical Trial
Source: JAMA Netw Open. 2024 May 16;7(5):e2410721. doi: 10.1001/jamanetworkopen.2024.10721 (PMC11099691; doi:10.1001/jamanetworkopen.2024.10721)
Supplement: Supplement 3. — Data Sharing Statement [file jamanetwopen-e2410721-s003.pdf]

Data Sharing Statement

Bieleninik. Music Therapy in Infancy and Neurodevelopmental Outcomes in Preterm Children. *JAMA Netw Open*. Published May 16, 2024. doi:10.1001/jamanetworkopen.2024.10721

Data
Data available: Yes
Data types: Deidentified participant data
How to access data: De-identified datasets (site codes, allocation, primary and secondary outcomes) are stored in a publicly available repository (https://osf.io/smjka/) and will be made available with publication.
When available: With publication

Supporting Documents
Document types: Statistical/analytic code
How to access documents: Statistical analysis scripts generated during the LongSTEP trial are stored in a publicly available repository (https://osf.io/smjka/) and will be made available with publication.
When available: With publication

Additional Information
Who can access the data: anyone requesting the data
Types of analyses: any purpose
Mechanisms of data availability: with investigator support
